# Normal sleep bouts are not essential for *C. elegans* survival and FoxO is important for compensatory changes in sleep

**DOI:** 10.1186/s12868-018-0408-1

**Published:** 2018-03-09

**Authors:** Heather L. Bennett, Yulia Khoruzhik, Dustin Hayden, Huiyan Huang, Jarred Sanders, Melissa B. Walsh, David Biron, Anne C. Hart

**Affiliations:** 1 Department of Molecular Biology, Cell Biology and Biochemistry, Brown University, 185 Meeting Street, Providence, RI 02912 USA; 2 Department of Neuroscience, Brown University, 185 Meeting Street, Box GL-N, Providence, RI 02912 USA; 3 Department of Pediatrics, Washington University School of Medicine, St. Louis, MO 63110 USA; 4 Department of Physics, Institute for Biophysical Dynamics, and James Franck Institute, The University of Chicago, 929 E. 57th St., Chicago, IL 60637 USA

**Keywords:** Compensatory sleep, *daf*-*16*, *lag*-*2*, Notch, Homeostasis, *jnk*-*1*, Mechanical stress

## Abstract

**Background:**

Sleep deprivation impairs learning, causes stress, and can lead to death. Notch and JNK-1 pathways impact *C. elegans* sleep in complex ways; these have been hypothesized to involve compensatory sleep. *C. elegans* DAF-16, a FoxO transcription factor, is required for homeostatic response to decreased sleep and DAF-16 loss decreases survival after sleep bout deprivation. Here, we investigate connections between these pathways and the requirement for sleep after mechanical stress.

**Results:**

Reduced function of Notch ligand LAG-2 or JNK-1 kinase resulted in increased time in sleep bouts during development. These animals were inappropriately easy to arouse using sensory stimulation, but only during sleep bouts. This constellation of defects suggested that poor quality sleep bouts in these animals might activate homeostatic mechanisms, driving compensatory increased sleep bouts. Testing this hypothesis, we found that DAF-16 FoxO function was required for increased sleep bouts in animals with defective *lag*-*2* and *jnk*-*1*, as loss of *daf*-*16* reduced sleep bouts back to normal levels. However, loss of *daf*-*16* did not suppress arousal thresholds defects. Where DAF-16 function was required differed; in *lag*-*2* and *jnk*-*1* animals, *daf*-*16* function was required in neurons or muscles, respectively, suggesting that disparate tissues can drive a coordinated response to sleep need. Sleep deprivation due to mechanical stimulation can cause death in many species, including *C. elegans*, suggesting that sleep is essential. We found that loss of sleep bouts in *C. elegans* due to genetic manipulation did not impact their survival, even in animals lacking DAF-16 function. However, we found that sleep bout deprivation was often fatal when combined with the concurrent stress of mechanical stimulation.

**Conclusions:**

Together, these results in *C. elegans* confirm that Notch and JNK-1 signaling are required to achieve normal sleep depth, suggest that DAF-16 is required for increased sleep bouts when signaling decreases, and that failure to enter sleep bouts is not sufficient to cause death in *C. elegans*, unless paired with concurrent mechanical stress. These results suggest that mechanical stress may directly contribute to death observed in previous studies of sleep deprivation and/or that sleep bouts have a uniquely restorative role in *C. elegans* sleep.

**Electronic supplementary material:**

The online version of this article (10.1186/s12868-018-0408-1) contains supplementary material, which is available to authorized users.

## Background

The timing of sleep is regulated by two semi-independent mechanisms that determine the propensity to sleep [1, 2]. Circadian rhythms regulate sleep synchrony with light/dark cycles and numerous conserved proteins critical for this complex pathway has been described. Homeostatic mechanisms regulate sleep drive, forcing compensatory increases in sleep quantity or depth after prolonged wakefulness, and facilitating arousal after restorative sleep [3]. Homeostatic sleep has been described in vertebrates and invertebrates using behavioral criteria, suggesting that the underlying regulatory mechanisms are also conserved across species. Yet, the precise molecular pathway or pathways that first respond to sleep deprivation and then drive compensatory sleep are still under investigation.

*Caenorhabditis elegans* sleep shares many of the behavioral features characteristic of mammalian sleep. These features include cessation of movement during sleep bouts, along with decreased responsiveness to mechanical or other sensory stimuli [4]. Many *C. elegans* studies focus on sleep occurring during lethargus, a developmental transition at the end of each larval stage. Lethargus lasts almost 3 h. During this period there are overt developmental and morphological changes, including vulval eversion, cuticle shedding/remodeling, as well as regulated seam cell and hypodermal/cuticle cell divisions [5]. These physical changes are temporally coordinated with other changes. During lethargus, *C. elegans* stop feeding, based on lack of pharyngeal pumping. Pharyngeal muscle intrinsic excitability is profoundly decreased during lethargus [6]. Most strikingly, during lethargus animals spontaneously enter into overt sleep bouts, which can last for several minutes that are characterized by complete lack of movement. Sleep bouts are characterized by increased time to move in response to external stimulation, which corresponds to an increased arousal threshold [7, 8]. Lethargus usually ends with vulval eversion and the establishment of adult behavior. Here, we focus on sleep bouts during *C. elegans* lethargus as only during these transient bouts are the behavioral changes characteristic of sleep observed (altered locomotion, posture, and arousal threshold).

Conserved proteins and pathways regulate sleep in animals; virtually all of the genes that regulate sleep in *Drosophila* also regulate sleep in *C. elegans,* including genes in the Notch pathway [8]. Perturbation of the *C. elegans* Notch signaling alters the sleep quantity and depth of sleep during the last larval diapause [7], but these changes are complex. Increased Notch signaling increases sleep bout quantity and results in deeper sleep, based on increased response time. But, modestly reduced Notch signaling results in increased sleep with decreased response time only during sleep bouts, which may signify poor quality sleep [7]. This combination of increased sleep with decreased response time only during sleep bouts is rare in the *C. elegans* literature. To our knowledge, only animals with decreased *c*-*Jun N*-*terminal kinase* (*jnk*-*1*) activity have a similar constellation of defects. JNK-1 is a conserved activator of stress responses across species; loss of *jnk*-*1* also results in low arousal thresholds during sleep bouts and increased time in sleep bouts [8]. The increase in sleep caused by reduced *C. elegans* Notch or JNK-1 signaling could reflect activation of homeostatic response that could compensate for poor quality sleep by driving increased or prolonged sleep bouts. Here, we examine this hypothesis.

Across the animal kingdom, modest sleep deprivation results in behavioral compensation including accelerated resumption of sleep (decreased sleep latency), deeper sleep (increased response time) and/or rebound sleep (extended time asleep). Physiological changes are also driven by sleep deprivation. The most profound reported consequence of prolonged sleep deprivation is death. Classical experiments demonstrated that prolonged, enforced sleep deprivation results in death within weeks or months, in both vertebrate and invertebrate species [9, 10]. Mechanical perturbation is used to wake animals in these studies. To control for the potential damaging stress of mechanical stimulation, control animals are either subjected to mechanical stress during the day or yoked animals are subjected to the same mechanical stress regardless of waking/sleep status. Control and yoked animals survive longer than animals deprived of sleep, which is consistent with a special requirement for sleep under these conditions. The molecular pathways required for survival after mechanical perturbation, in sleep or motion bouts, have not been carefully examined. Here, we focus on the role of DAF-16 FoxO, a FoxO transcription factor critical for stress resistance in many contexts.

Previous work suggested that FoxO has little impact on sleep bouts in unperturbed young *C. elegans* under standard conditions [11–13]. FoxO is required for homeostatic/compensatory changes seen in *C. elegans* subjected to force waking from sleep bouts during the last larval lethargus [11, 12]. Counter-intuitively, in *Drosophila* loss of *FoxO* function increases sleep in aged animals that are also defective in insulin signaling [13]. Additionally, both FoxO signaling and sleep duration in *C. elegans* are impacted by food availability [11, 12, 14]. Therefore, impact of FoxO on sleep in invertebrate models is potentially complex.

Here, we examine the role of DAF-16 FoxO in three different sleep paradigms: altered Notch signaling, altered JNK-1 kinase signaling, and mechanical stimulus-induced sleep perturbation. Our results demonstrate that DAF-16 FoxO is required for sleep bouts changes in all three paradigms. However, arousal threshold changes are not dependent on DAF-16 function. We confirm that normal sleep bouts are not required for *C. elegans* survival, but find that sleep bouts are important when animals are subjected to mechanical stress, a result that has implications for studies of sleep deprivation in other organisms.

## Methods

### Strains used in study

N2 referred to as wild type [15]; *daf*-*16(mgDf50), lin*-*12(n941)*, *dsl*-*1(ok810)* and *jnk*-*1(gk7)* are null alleles. CF1038 *daf*-*16(mu86lf) I*, GR1307 *daf*-*16(mgDf50)* JK1277 *lag*-*2(q420tslf) V*, GS3662 *dsl*-*1(ok810) IV*, MT688 *lin*-*12(n137n460csgf) III*, HA2518 *uIs72[myo*-*2p::RFP, unc*-*119p::sid*-*1;unc*-*119p::mec*-*18];sid*-*1(*+*)* generated from *TU3595* by backcross into N2 3 times, eliminating *lin*-*15(n765)* [16], HA1464 *lin*-*12(n941)/qC1 [rol*-*6(d),lag*-*2p::gfp] III,*CF1903 *glp*-*1(e2141lf) III,* VC8 *jnk*-*1(gk7) IV,*VC20318 *jip*-*1(rt249lf) II,* VC40349 *jip*-*1(lrt250f) II,* CF1880 *daf*-*16(mu86lf) I;glp*-*1(e2141lf) III,* HA2776 *daf*-*16(mu86lf)I;lin*-*12(n137n460csgf) III,* HBR227 *aptf*-*1(gk794lf) II,* HA1863 *osm*-*7(tm2256) III; osm*-*11(rt142) X,* HA1133 *rtIs26[hsp::osm*-*11, elt*-*2p::gfp, pha*-*1(*+*)] III.* GR1395 *mgIs49[mlt*-*10p::gfp*-*PEST, ttx*-*3p::gfp] IV,* HA2526 *mgIs49[mlt*-*10p::gfp*-*PEST, ttx*-*3p::gfp] IV; lag*-*2(q420tslf) V,* NQ440 *daf*-*16(mgDf50)I;qnIs42 [Punc*-*119:gfp::daf*-*16;Pmyo*-*2:mCherry]* from Driver et al. 2013 [11]. NQ441 *daf*-*16(mgDf50) I;qnIs45[Pdaf*-*16;gfp::daf*-*16;Pmyo*-*2:mCherry]* from Driver et al. 2013 [11]. NQ145 *daf*-*16(mgDf50) I;qnEx38[Pmyo*-*3:gfp::daf*-*16;Pmyo*-*2:mCherry]* from Driver et al. 2013 [11], HA2527 *daf*-*16(mu86lf) I;lag*-*2(q420tslf) V,* HA2727 *daf*-*16(mgDf50) I;lag*-*2(q420tslf) V,* HA2728 *jnk*-*1(gk7)IV;lag*-*2(q420tslf) V*, HA2740 *daf*-*16(mgDf50)I;jnk*-*1(gk7) IV*, HA2755 *daf*-*16(mgDf50) I;jnk*-*1(gk7) IV;lag*-*2(q420tslf) V*, HA2756 *daf*-*16(mu86lf) I;lin*-*12(n941)/qC1 III,* HA2757 *daf*-*16(mgDf50) I;aptf*-*1(gk794lf) II*, HA2758 *daf*-*16(mgDf50) I;qnIs45[Pdaf*-*16;gfp::daf*-*16;Pmyo*-*2:mCherry];aptf*-*1(gk794lf) II,* HA2760 *daf*-*16(mgDf50) I;qnIs42[Punc*-*119:gfp::daf*-*16;Pmyo*-*2:mCherry];lag*-*2(q420tslf) V*, HA2761 *daf*-*16(mgDf50) I; qnEx38[Pmyo*-*3:gfp::daf*-*16;Pmyo*-*2:mCherry];lag*-*2(q420tslf) V,* HA2762 *daf*-*16(mgDf50) I;qnIs42[Punc*-*119:gfp::daf*-*16;Pmyo*-*2:mCherry];jnk*-*1(gk7) IV,* HA2763 *daf*-*16(mgDf50) I;qnEx38[Pmyo*-*3:gfp::daf*-*16;Pmyo*-*2:mCherry];jnk*-*1(gk7) IV. C. elegans* used in the study were obtained from *Caenorhabditis* Genetics Center (CGC). Other strains were generated from these as described.

### *Caenorhabditis elegans* culture

Strains were reared on NGM plates seeded with OP50 *E. coli* under standard conditions [15]. *lag*-*2(q420tslf)* is a temperature sensitive, loss of function allele [17, 18]. Adult hermaphrodites were reared at 15 °C, shifted to 25.5 °C and their F1 progeny were assayed for behavioral defects, unless otherwise indicated in temperature-shift studies. *lin*-*12(n137n460csgf)* is a cold sensitive, gain of function allele [19]. Animals were reared at 15 °C and assayed at 22 °C for total sleep. Other strains were reared at 25 °C and assayed at 22 °C.

### Sleep assays

Sleep and motion bouts were assayed during the L4-to-adult lethargus, unless otherwise stated. L4 stage animals were loaded into 1 × 4 mm microfluidic chambers and assayed for total time in sleep bouts or motion bouts, based on movement/immobility. As previously described in Singh *et al.* [7], bouts were measured based on consecutive image subtraction using a custom Matlab program. Camera equipment included: AxioCam ICc1 (Zeiss, Oberkochen, Germany) pixel size 4.65 μm × 4.65 μm. AxioCam MRc Rev3 (Zeiss) pixel size 6.45 μm × 6.45 μm. Stingray F201c (Allied Vision Technologies, Stadtroda, Germany) pixel size 4.4 μm. We report here that food density can affect sleep bout quantity; see Supplement for details.

Total time in sleep bouts was calculated using images taken every 10 s over 12 h, spanning the approximately 2.5 h of lethargus. Occasionally, animals were damaged during chamber loading and were inappropriately motionless in the L4 stage; these were censored. A rolling average over 60 images was used for fractional sleep/quiescence calculations. Lethargus entry was defined as the time point when fractional sleep/quiescence (fQ) exceeded 0.1 and lasted for at least 20 min. Lethargus entry is the first sleep bout in that 20-min window. Lethargus exit was defined as the time when fQ remained below 0.1 for at least 20 min. Lethargus exit is the last sleep bout before that 20 min window. For analysis of sleep, lethargus was arbitrarily divided into four 45-min periods, referred to as stages I, II, III, IV, and A (for the 45 min after lethargus). Bouts spanning two stages of lethargus are included in both stages. As a result, the number of bouts used in this calculation may exceed the number of bouts in lethargus. Vulval eversion usually occurs in stage IV, but sleep bouts were observed after vulval eversion (based on transient lack of motion) in specific mutant strains. We did not independently score vulval eversion or cuticle shedding as measures of developmental diapause duration.

For Additional file 1: Fig. 2, Motion and quiescence were identified similarly using the image difference method as described in Nagy *et al.* [20]. The total time spent in quiescence was calculated as the sum of durations of all bouts of quiescence.

### Arousal threshold

Mechanosensory response to body touch was used to assess response time. NGM plates were seeded with a 100 μl of OP50 bacteria and allowed to dry at room temperature overnight. A thin hair was placed on the NGM plate and the hair was flexed to contact the sleeping animal behind the pharynx, with an estimated force less than 10 μN [21]. Animals that immediately initiated backward locomotion were scored as responding; percent responding animals is reported. To detect arousal threshold differences throughout the entire L4 to adult molt, lethargus was arbitrarily divided into five 45-min periods referred to as I, II, III, IV, and V stages. Twenty animals were selected for entry into the L4 lethargus based on vulval morphology and arousal threshold was measured during each stage randomly selecting ten of these animals. Arousal threshold results were determined in at least 3 independent trials with an n = 10 for each genotype tested. At least one trial was undertaken blinded, blinded trials agreed with non-blinded trails and are explicitly stated in the supplementary materials and figure legends. This approach was used for all arousal threshold determinations. None of the genotypes examined here are defective or hypersensitive in response to touch outside of lethargus or during motion bouts.

### Heat-shock notch ligand transgene induction

Transgenic animals were generated by using standard strategies [22]. pPD49.26 plasmid containing *hsp::lag*-*2cDNA* was injected at 100 ng/μl together with pCFJ90 *myo*-*2p::mCherry,* at 5 ng/μl, and pBX#1 plasmid containing a *pha*-*1* rescue construct, at 150 ng/μl, into *pha*-*1(e2123lf) III* animals. Control animals were generated by injecting pPD49.78 containing nothing (*hsp::empty promoter*), at 100 ng/μl, pCFJ90 at 5 ng/μl, and pBX#1 at 150 ng/μl into *pha*-*1(lf)* animals. To induce expression of *lag*-*2* expression young adult animals were transferred to 10 ml NGM plates seeded with 200 μl of thinly spread OP50, sealed with Parafilm, and floated in a water bath at 34 °C for 75 min. Before scoring, animals were transferred to 20 °C for 60 min for recovery from heat shock, blinded as to genotype whenever possible. The duration of heat shock and 60 min recovery period is similar to or shorter than protocols reported previously [7]. Sleep was scored as the absence of pharyngeal pumping and locomotion for more than 5 s.

### Temperature shift experiments

Downshift: *lag*-*2(q420tslf)* animals were raised at the permissive temperature of 15 °C. Adult hermaphrodites were temperature shifted to NGM plates seeded with 200 microliters (μl) of OP50 *E. coli*, from the permissive temperature and allowed to lay eggs at the restrictive temperature. The F1 progeny were raised at 25 °C and then shifted to 15 °C as early L4 stage larvae. Sleep assays were started 4 h after shifting animals to 15 °C to capture the entire L4 lethargus. Upshift: *lag*-*2(q420tslf)* adult hermaphrodites were raised at 15 °C and allowed to lay eggs. Progeny were raised at 15 °C and temperature shifted to 25 °C as early L4 stage larvae. 12 h videos were recorded and total time in sleep bouts during the L4 to adult molt was calculated. We found that when L4 stage *lag*-*2(q420tslf)* animals reared at 15 °C were kept at room temperature for more that 20 min, there was a perceptible increase in sleep bout number during L4/A lethargus. Similarly, downshift to room temperatures of 20 °C modestly decreased L4/A sleep bouts. Therefore, we minimized exposure to room temperature for *lag*-*2(q420tslf)* animals in all studies.

### RNAi by feeding

Wild type (N2) or animals expressing the double stranded RNA channel SID-1 in neurons [16] were reared on either empty, control pL4440 or *lag*-*2(RNAi)* or *jnk*-*1(RNAi)* clones to knockdown transcripts. Animals were reared for two generations on RNAi plates before testing behavior. All RNAi clones were confirmed by sequencing. *jnk*-*1(RNAi)* plasmid pHA#667 [8] contains 1.8 kb of *jnk*-*1* PCR amplified genomic sequence including exons cloned into pL4440 using NheI and SacI sites. *cya*-*1(RNAi)* plasmids pHA#662 and pHA#663 [8]: non-overlapping, PCR-amplified *cya*-*1* cDNA sequences were cloned into NheI and XmaI sites of pL4440. The *lag*-*2(RNAi)* clone was obtained from the Vidal feeding clone library [23]. The relative level of knockdown was not measured. *C. elegans* tissues vary dramatically in their response to RNAi by feeding and neurons are particularly insensitive.

### Acute hypertonic stress

Young adult animals were reared at 25 °C and tested on 500 mM NaCl NGM plates with no OP50 bacteria. Animals were left on plates for 10 min and scored for the number of animals spontaneously moving *versus* immotile. This approach was only used in Additional file 2: Table 1.

### Vulval development, diapause duration, and staging

N2 and *lag*-*2(tslf)* animals were raised at 15 °C. Adult hermaphrodites were shifted to 25 °C where animals were allowed to lay eggs. The F1 progeny were selected at the mid- L4 stage and transferred to seeded NGM plates. Animals were placed at room temperature and the percent of animals with appropriate vulval eversion was recorded 3 h later. None of the *lag*-*2(q420)* animals had protruding vulvae in these experiments.

### Rescue using neuronal promoter

A 3.5 kb region of the *aex*-*1* promoter was excised from pPD49.26 using NheI and NcoI restriction enzymes. *jnk*-*1 cDNA* was PCR amplified using forward primer gaacgctagcATGGAGGAACGATTATCCAC and reverse primer gaacCCATGGTCAGGAATAAATGTCATGGG and ligated into pPD49.26.

### Mechanical stress assay

Experimental design was adapted from a previous study [11]. Animals were selected at the beginning of the L4 lethargus. To induce sleep deprivation, animals were physically stimulated in the tail region using a platinum wire when locomotion paused. These animals are referred to as “poked”. Yoked animals were physically stimulated with the same force whenever poked animals were prodded, regardless if in sleep or motion bout. The stimulation period lasted on average 40 min; stimulation ended when poked animals no longer responded to stimulus. Animals that burst at the vulva were censored. 24 h after stimulation, the number of dead adult animals (neither moving nor feeding when prodded) and living animals was assessed. These studies were undertaken by an experimenter naïve as to predicted outcomes and hypotheses. This procedure was used only in Fig. 6b and Additional file 3: Table 2.

### Sleep disruption assay

To disrupt sleep, vibrations (1 kHz) were delivered as described in Nagy *et al.* [12]. In brief, an electronic buzzer was mechanically coupled to the plate containing the worms using an acrylic clamp and controlled using Matlab (Mathworks Inc., Natick, MA). Ten second square wave stimuli were delivered every 10 min for 10 h that included the period of L4 lethargus, regardless of sleep/motion bout status. This procedure was used only in Additional file 1: Fig. 2.

### Statistical analysis

Student’s two tailed t-test was used for comparisons of sleep metrics between two genotypes/treatments and for rescue experiments. When more than three significance determinations were required for multiple comparisons for sleep metrics, a Bonferroni correction was applied. In arousal studies testing double mutant animals for suppression/synergy, we first determined when the single mutant animals differed from wild type using one way ANOVA and Tukey post hoc-tests. Then, only at time points where the single mutant animals differed from wild type, we determined if the double mutant animals suppressed/synergized with the single mutants using a student’s two tailed t-test. Chi square analysis was used for survival in mechanical stress studies. All experimental results, including number of animals scored, blinded trials, and number of independent replicates is available in Supplementary Materials. All statistical analysis and graph construction were prepared using GraphPad Prism version 7.

## Results

### Decreased *lag*-*2* function resulted in increased sleep bouts with decreased arousal thresholds

To understand the relationship between sleep, arousal, and sleep homeostasis, we re-examined the impact of Notch signaling on *C. elegans* developmental sleep, focusing on the role of *C. elegans* DSL ligand LAG-2. In this analysis, we examine sleep bouts during the last *C. elegans* larval diapause, *i.e*. during the transition from the L4 larval to adult stage. L4/A lethargus sleep bouts fit the behavioral definition of sleep, including decreased behavioral response to sensory stimulation, specific posture, transient and spontaneous immobility, as well as homeostatic regulation after sleep bout disturbance [2]. Sleep bouts were measured herein using previously described tracking approaches, which discriminate between sleep bouts and motion bouts based on locomotion/immobility [7, 20, 24, 25].

**Fig. 1:**
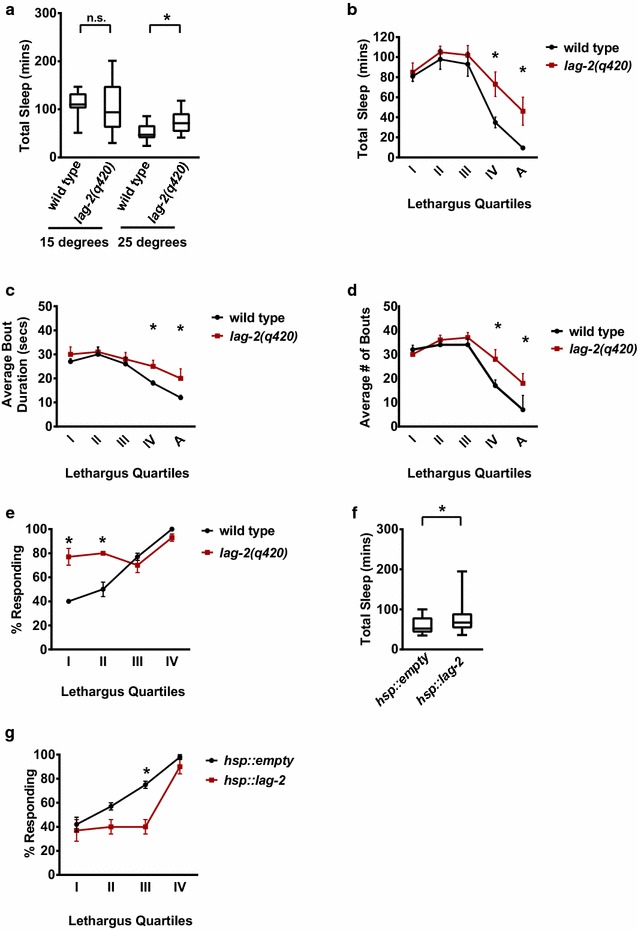
Notch ligand *lag*-*2* regulates sleep bout quantity and arousal threshold during L4/A lethargus. **a** Partial loss of *lag*-*2* resulted in increased sleep. Box shows two middle quartiles, horizontal line indicates mean, bars represent maximum/minimum; * denotes statistical significance with p ≤ 0.01 *versus* wild type by student’s two tailed t-test. n = 13, except 14 for *lag*-*2(q420)* at 15 °C. **b** Partial loss of *lag*-*2* increases sleep during the final stages of lethargus. Time in sleep bout shown during L4/A lethargus, split into 45 min intervals/quartiles: I, II, III, IV, and “After”. Significance *p ≤ 0.05 for wild type *versus lag*-*2(q420)*; other time points not significant based on multiple t-tests comparison followed by a Bonferroni correction; error bars represent SEM. Four independent trials combined; 40 animals per genotype for each time point. **c** Loss of *lag*-*2* function increased average pause duration in the last stages of lethargus. Significance and analysis indicated as in panel B. n = 13 for both genotypes. **d** Partial loss of *lag*-*2* increases sleep bout frequency during final stages of L4/A lethargus. Significance and analysis indicated as in Panel B. **e** Decreased *lag*-*2* function increased time in sleep bouts. Percent sleeping animals responding during 45 min intervals sequentially named I, II, III, and IV. Four independent trials combined here, totaling 40 animals per genotype at each time point. p ≤ 0.001 assessed as in panel B; error bars represent SEM. **f** Over-expression of *lag*-*2* increased time in sleep bouts. *hsp::lag*-*2* is leaky even at room temperature. Significance as in Panel A. *hsp::empty* n = 23, hsp*::lag*-*2* n = 25. **g** Over-expression of LAG-2 resulted in increased arousal thresholds during sleep bouts. Animals carry *hsp::empty* or *hsp::lag*-*2* transgenes. Significance as in Panel B. Results from three independent trials reported, 30 animals total each

Perturbation of Notch signaling disrupts L4/A lethargus sleep bouts in a previously described complex allelic series [7]. Complete loss of *lag*-*2* function is lethal in embryonic stages; here we primarily use the temperature-sensitive, partial loss-of-function allele, *lag*-*2(q420tslf)*. When reared and tested at the permissive temperature of 15 °C, where normal LAG-2 function is expected, *lag*-*2(q420tslf)* animals total time in sleep bouts during the L4-to-adult (L4/A) lethargus was indistinguishable from that of wild type animals (Total sleep, Fig. 1a, Additional file 4: Fig. 1A N2 used as wild type *C. elegans* laboratory strain). *C. elegans* develop more quickly at 25 °C [15]. We found that total time in sleep bouts during L4/A decreased at warmer temperatures in well-fed, wild type animals, with decreased sleep bout duration and decreased lethargus duration. (Figure 1a, Additional file 4: Fig. 1A, B). However, *lag*-*2(q420tslf)* animals had increased total sleep (time in sleep bouts) during in late L4/A lethargus, compared to control wild type animals. During early stages of L4/A lethargus, total time in sleep for wild type and *lag*-*2(q420tslf)* animals were similar; differences arose late in lethargus (Fig. 1b–d and Additional file 5: Raw Data File). We found that loss of another *C. elegans* DSL ligand, *dsl*-*1*, had no impact on sleep bouts (Additional file 4: Fig. 1C), but increased time in sleep bouts has been reported previously for animals with decreased function in Notch receptor or co-ligands [7]. Thus, LAG-2 Notch ligand function is required for normal sleep bout quantity during the transition from the last larval stage to adulthood in well-fed animals; decreased function leads to increased sleep bouts late in L4/A lethargus and, hence, prolonged behavioral lethargus.

Over the course of our studies, we noted that the availability of food had a significant impact on sleep bouts during L4/A lethargus. For work presented here, late L4 stage larvae were moved from plates with plenteous *E. coli* food to microfluidic assay chambers with various concentrations of *E. coli*. Increased food concentrations led to increased total time in sleep bouts during L4/A lethargus in wild type animals (Additional file 1: Fig. 2). Previous work has demonstrated that stress can induce a sleep-like state that increases survival of adult *C. elegans* [26–29]. A recent study demonstrated that *C. elegans* alter behavior in response to environmental stimuli and food availability [30]. We tentatively suggest the decreased sleep bout number observed at lower food concentrations may involve a stress response. As DAF-16, JNK-1 and LAG-2 signaling pathways have previously been implicated in various stress responses; we opted to diminish the possible impact of food-restriction on sleep bouts by undertaking all subsequent assays in this manuscript at higher concentrations of *E. coli*, which yields the same quantity of L4/A sleep bouts as observed in *C. elegans* on standard culture plates with plenteous food. The impact of feeding on sleep bouts and response to sleep deprivation is of considerable interest, will likely be complex, may be genotype dependent, and will be considered in future studies.

Increased arousal thresholds during sleep are a signal feature of sleep across species; aberrantly decreased arousal thresholds during sleep are usually associated with shallow, less restorative sleep. Perturbation of Notch signaling decreases arousal thresholds during *C. elegans* sleep bouts. For example, animals with reduced activity in Notch receptors have inappropriately increased response to external stimulation during L4/A sleep bouts, compared to controls [7]. However, their response during lethargus motion bouts or as adult animals is equal to control animals, consistent with a selective defect in sleep depth during lethargus sleep bouts in animals with decreased Notch signaling. To detect arousal threshold differences during L4 to adult molt, lethargus was arbitrarily divided into five 45-min periods referred to as I, II, III, IV, and V stages. Gentle touch with a hair was used to wake animals from sleep bouts. During early lethargus time points, wild type animals in sleep bouts responded to gentle touch at relatively low rates. During sleep bouts at early stages, *lag*-*2(q420)* animals had inappropriately high response rates to mechanical stimulation (Fig. 1e). Later in lethargus, response rates increased in wild type animals, approaching the response rate of adult animals or L4/A animals in motion bouts (Fig. 1e). And, at these later time points, the response rates of sleeping *lag*-*2(q420)* animals were similar to control animals; even though sleep bout durations are equal to or exceed those of control animals. We found that *lag*-*2(q420)* mutant and wild type responses to touch were indistinguishable, both during L4/A lethargus motion bouts and during adult stages. This rules out a defective sensory response, per se and suggests that sleep bouts may be specifically affected by altered Notch signaling. We conclude that, in the presence of plenteous food, decreased *lag*-*2* function results in decreased arousal thresholds during sleep bouts and increased sleep quantity.

To confirm these behavioral changes were due to altered activity of the Notch ligand LAG-2, we examined the consequences of manipulating *lag*-*2* expression during lethargus. LAG-2 expression was increased using a transgene, *hsp::lag*-*2* that places expression of the *lag*-*2* cDNA under the control of the *C. elegans hsp*-*16* promoter. The *hsp*-*16.2* promoter fragment used in the transgene is known to be “leaky” and modest expression occurs at 25 °C, while heat shock dramatically increases expression (described below). On an otherwise normal background and without heat shock induction, the *hsp::lag*-*2* transgene modestly increased total time in sleep bouts during L4/A lethargus, relative to control animals that carried the same transformation markers and an empty *hsp*-*16* transgene lacking *lag*-*2* sequences (Fig. 1f). Also, we found that this increased LAG-2 expression resulted in increased response times during L4/A lethargus sleep bouts (gentle touch, no heat shock Fig. 1g). These results are consistent with previous findings: increased Notch activity results in increased response time specifically during sleep bouts and increased time asleep during the L4/A lethargus [7]. Combined, these results confirm that LAG-2 regulates sleep bout quantity and arousal thresholds during developmentally-timed sleep bouts during *C. elegans* L4/A lethargus.

### *lag*-*2* does not impact lethargus duration and sleep via cell fate changes

Notch signaling has diverse roles in cell fate specification and proliferation during development. For example, altered *lag*-*2* function during larval stages causes cell fate specification changes resulting in vulval defects [31]. This raised the possibility that decreased *lag*-*2* function might alter L4/A lethargus sleep bouts due to changes in developmental timing or cell fate specification. We found that, based on previously established behavioral metrics, lethargus entry was not delayed in *lag*-*2(q420)* animals. However, L4/A behavioral lethargus exit was delayed based on locomotion tracking, resulting in increased lethargus duration (Additional file 4: Fig. 1). The duration and frequency of sleep bouts in control animals and *lag*-*2(q420)* animals was roughly similar during L4/A lethargus, although average sleep bout duration in *lag*-*2(q420)* animals was consistently longer, especially in later stages of lethargus (Additional file 4: Fig. 1A). However, the most striking difference between *lag*-*2(q420)* and control animals was extended lethargus duration (as defined by sleep bouts). In control animals, vulval eversion was coincident with the disappearance of sleep bouts. By contrast, in *lag*-*2(q420)* animals, sleep-like bouts with transient cessation of feeding were observed for a very short time after vulval eversion. Increased behavioral lethargus duration has been reported previously for animals with decreased function in Notch receptor or co-ligands [7], but the timing of vulval eversion is also not altered in these animals suggesting that diapause and overall development are not affected. Importantly, we did not observe sleep-like bouts before L4/A lethargus in *lag*-*2(q420)* animals nor were they observed more than an hour after vulval eversion, suggesting that defect specific to lethargus. *lag*-*2(q420)* locomotion levels are not globally decreased; at restrictive temperatures activity is increased in adult animals (Table S1B in Singh et al. [7]). To assess if overall developmental timing was affected in *lag*-*2(q420)* animals, we monitored the timing of vulval development. We selected animals at the vulval “Christmas tree stage”, which corresponds to L4 lethargus entry [32]. After 3 h, we determined the fraction of animals reaching adulthood, based on successful vulval eversion. Similar fractions of wild type and *lag*-*2(q420)* animals reached this developmental landmark after 2 h, which is coincident with entry into adulthood (Additional file 6: Fig. 3). We also noted that it was much easier to find *lag*-*2(q420)* animals in sleep-like bouts within an hour after vulval eversion, compared to wild type animals. Interestingly, after vulva eversion, response to mechanosensory stimulation was robust in *lag*-*2(q420)* animals, suggesting that response time is not increased during these apparent sleep bouts. These results suggest that *lag*-*2(q420)* disrupts the quantity and timing of sleep and sleep-like bouts, but does not change the overall timing of diapause and the transition to adulthood.

**Fig. 2:**
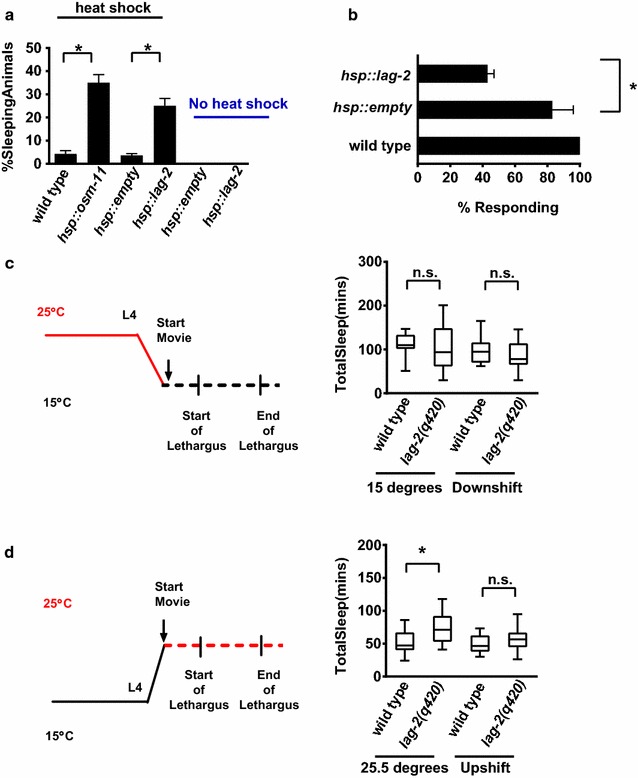
LAG-2 regulation of L4/A lethargus sleep cannot be ascribed to cell fate changes. **a** Over-expression of LAG-2 in adult animals induces inappropriate sleep bouts. Animals were heat shocked and percentage displaying inappropriate sleep was determined after a 1 h recovery period at 20 °C. * denotes statistical significance at p < 0.001 *versus hsp::empty* based on student’s two tailed t-test; error bars represent the standard error of the mean. **b** Adults in inappropriate sleep bouts had increased arousal thresholds. Percent response of sleeping animals is reported for two independent trials, n = 20 animals in total for each genotype. * denotes statistical significance, determined as in panel A, of p = 0.02 *versus hsp::empty*; error bars represent the standard error of the mean. **c** Increasing *lag*-*2* activity before and during L4/A lethargus restored normal sleep quantity. Animals raised at the restrictive temperature of 25.5 °C were shifted to 15 °C 4 h prior to L4 lethargus (illustration, left side of panel) *lag*-*2(q420)* is a temperature-sensitive, loss of function allele with decreased function at higher temperatures. Lack of statistical significance based on student’s two tailed t-test. Wild type n = 13 animals and *lag*-*2(q420)* n = 14 at 15 °C; wild type n = 15 animals and *lag*-*2(q420)* n = 19 at 25.5 °C. **d** Decreasing *lag*-*2* activity before and during L4/A lethargus did not impact sleep quantity. Animals raised at the permissive temperature of 15 °C were shifted to the restrictive temperature of 25.5 °C for 4 h prior to L4 lethargus (illustration, left side of panel). Total time in sleep bouts is reported in minutes as in panel C. Wild type n = 13 *lag*-*2(q420)* n = 14 at 15 °C, wild type n = 10 and *lag*-*2(q420)* n = 14 at 25.5 °C. Statistical significance determined as in panel A

We also considered the possibility that altered cell fate decisions during the development of *lag2(q420)* animals might drive behavioral changes during lethargus. If so, then increasing *lag*-*2* activity only in adult animals would be unlikely to induce anachronistic sleep bouts. Previous work has demonstrated that transient over-expression of the Notch DOS co-ligand OSM-11 in young adult *C. elegans* results in anachronistic adult sleep bouts [7]. We found that heat shock induction of *hsp::lag*-*2* expression similarly induced anachronistic sleep bouts in adult animals (Fig. 2a), even after disappearance of heat-shock stress-induced sleep in control animals. As expected, this anachronistic *hsp::lag*-*2*-induced sleep was characterized by the cessation of locomotion, complete inhibition of pharyngeal pumping, and decreased rate of response to touch stimuli, consistent with increased response time during sleep bouts (Fig. 2b). *hsp::lag*-*2* anachronistic sleep was reversible; 2 h after heat shock, animals resumed feeding and locomotion at control levels (Additional file 7: Fig. 4). The anachronistic sleep bouts observed in *hsp::lag*-*2* animals suggest that *lag*-*2* expression is sufficient to induce sleep bouts in this context and consistent with a role for Notch signaling during L4/A lethargus sleep, beyond developmental timing and cell fate specification.

If LAG-2 and Notch signaling regulate sleep bouts independent of cell fate specification, then manipulating Notch signaling before or during lethargus should alter L4/A sleep bouts. To confirm this and to determine when LAG-2 function is required, we took advantage of the temperature-sensitive nature of the *lag*-*2(q420)* allele. Animals were raised at the restrictive 25 °C temperature and switched to the permissive 15 °C temperature prior to L4/A lethargus, or vice versa. We found that when *lag*-*2(q420)* animals were shifted from the restrictive to the permissive temperature during early L4 larval stage, total sleep bouts in the L4/A were similar to wild type animals (Fig. 2c). This indicates that restoring LAG-2 activity just before or during the L4/A lethargus is sufficient to allow normal sleep bout number and that earlier cell fate perturbations due to decreased Notch signaling are likely not responsible for these behavioral defects. However, the reciprocal temperature shift did not induce an increase in total sleep bout number; *lag*-*2(q420)* animals shifted during early L4 larval stage from the permissive to restrictive temperature and wild type animals had similar numbers of sleep bouts (Fig. 2d). It seems likely that residual LAG-2 protein perdures; activated Notch receptors, and/or earlier gene expression from Notch transcriptional targets is likely sufficient to maintain normal sleep bout number during L4/A lethargus in the latter experimental paradigm. The *lag*-*2(q420)* allele affects a splice site; mRNAs may be accurately spliced at the permissive temperature [33]. Overall, these results suggest that *lag*-*2* function is required for normal sleep bout quantity and arousal thresholds during sleep bouts. This combination of defects (increased and prolonged sleep, but easy to rouse), is consistent with poor quality sleep driving increased sleep quantity by extension of lethargus duration.

### Decreased Notch signaling results in DAF-16 FoxO dependent increases in sleep

Singh et al. [7] reported previously that moderately decreased Notch signaling results in increased response to stimulation only during sleep bouts, with increased sleep quantity and increased lethargus duration for animals carrying a mutation in just one Notch pathway gene. It was suggested that poor quality sleep bouts might drive compensatory increases in sleep bout number and/or lethargus duration as a homeostatic response, reminiscent of compensatory sleep changes observed in mammals [34]. At the time, there was not a simple way to test this hypothesis. Subsequently, DAF-16, a FoxO transcription factor, was shown to be essential for homeostatic increases in *C. elegans* sleep bout quantity and depth after mechanical perturbation [11]. We examine here if DAF-16 is required for the increased sleep bouts observed in *lag*-*2(q420)* animals.

**Fig. 3:**
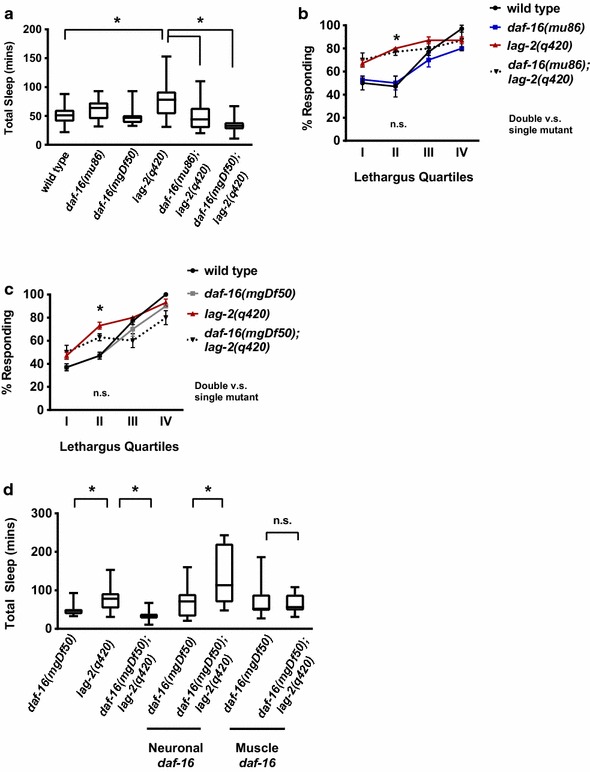
Alterations in *lag*-*2* Notch signaling disrupted sleep bouts during *C. elegans* L4/A lethargus. **a** Increased sleep in animals with decreased *lag*-*2* function was dependent on *daf*-*16* function. Parental animals were shifted from 15 °C to 25.5 °C. F1 progeny were selected at L4 stage and assayed at 25.5 °C. Box shows two middle quartiles, horizontal line indicates mean, bars represent maximum/minimum; * denotes statistical significance with p < 0.05 by student’s two tailed t-test. Wild type n = 26 animals, *daf*-*16(mu86)* n = 14, *lag*-*2(q420)* n = 33, *daf*-*16(mu86);lag*-*2(q420)* n = 14, *daf*-*16(mgDf50null)* n = 10, *daf*-*16(mgDf50null);lag*-*2(q420)* n = 15. **b** Arousal threshold defects caused by decreased *lag*-*2* function were not dependent on *daf*-*16* function. Percent response is indicated from at least four independent trials, n = 40 animals for each data point. Statistical significance was assessed by one way ANOVA followed by a Tukey post hoc-test; error bars respresent the SEM. *p < 0.05 for comparison of wild type *versus lag*-*2(q420) and* wild type *versus daf*-*16(mu86;lag*-*2(q420).* No statistical significance between *lag*-*2(420)* and *daf*-*16(mu86;lag*-*2(q420).* **c** Complete loss of *daf*-*16* function did not alter sleep bout arousal thresholds in animals with decreased *lag*-*2* function. Statistical analysis and presentation indicated as in Panel B. * p ≤ 0.05 for comparison of wild type and *lag*-*2(q420).* No statistical significance between *lag*-*2(420)* and *daf*-*16(mgDf50;lag*-*2(q420).* **d** In animals with decreased *lag*-*2* function, *daf*-*16* function was required in neurons for increased sleep. DAF-16 was expressed under the control of a neuron-specific or muscle-specific promoter in *daf*-*16(mgDf50) lag*-*2(q420)* animals. Statistical analysis and presentation represented as in Panel A. * indicates statistical significance with p ≤ 0.01 for comparisons to control. Animals tested: *daf*-*16(mgDf50)* n = 10, *lag*-*2(q420)* n = 33, *daf*-*16(mgDf50);lag*-*2(q420)* n = 15, *Punc*-*119: daf*-*16(mgDf50)* n = 14, *Punc*-*119:daf*-*16(mDf50);lag*-*2(q420*) n = 15, *Pmyo*-*3:daf*-*16(mgDf50)* n = 13, *Pmyo*-*3:daf*-*16(mgDf50);lag*-*2(q420)* n = 10

Animals were constructed carrying both *lag*-*2(q420)* and two alleles of *daf*-*16*: a partial loss of function allele, *daf*-*16(mu86),* and a complete loss of function, null allele, *daf*-*16(mgDf50)*. We confirmed that, in unperturbed animals, *daf*-*16* loss of function did not have a dramatic impact on sleep bout quantity during L4/A lethargus; total time spent in sleep bouts were roughly equal in *daf*-*16* loss of function and control animals at high food density (Fig. 3a, and Additional file 1: Fig. 2). However, under these conditions, loss of *daf*-*16* altered total time in sleep bouts for animals with decreased LAG-2 function. Decreased *daf*-*16* activity resulted in decreased total sleep bout number when *daf*-*16; lag*-*2(q420)* double mutant animals were compared to *lag*-*2(q420)* control animals. In animals carrying the partial loss of function allele *daf*-*16(mu86); lag*-*2(q420)*, sleep quantity returned to wild type levels (Fig. 3a). In animals carrying the complete loss of function allele *daf*-*16(mgDf50); lag*-*2(q420)*, total sleep bout number decreased to below wild type levels (Fig. 3a). In double mutant animals, neither sleep bout duration nor frequency was altered by decreased *daf*-*16* function (Additional file 5: Raw Data File); only lethargus duration was altered. We concluded that DAF-16 FoxO is required for the increased sleep observed in animals with diminished LAG-2 function.

Does loss of DAF-16 also restore normal response to stimulation during sleep bouts in lag-*2(q420)* animals? Our results suggest that this is not the case. Arousal thresholds during sleep bouts of *lag*-*2(q420)* animals were defective in lethargus stage II, which we defined as 45–90 min after the start of lethargus (Fig. 3b, c). Neither the partial loss of function allele *daf*-*16(mu86)* nor the null allele *daf*-*16(mgDf50)* affected on the response rates of *lag*-*2(q420)* animals during lethargus sleep bouts at this time point; double mutant animals responded at the same rates as *lag*-*2(q420)* animals (Fig. 3b, c). We conclude that loss of *daf*-*16* does not impact arousal thresholds in this context, but *daf*-*16* is required for increased sleep bouts in *lag*-*2(q420)* animals.

We next determined where *daf*-*16* function is required for increased sleep when Notch signaling decreased. We obtained previously characterized transgenic strains in which DAF-16 is expressed under the control of either a pan-neuronal promoter or a muscle-specific promoter [11, 35]. Each transgene was crossed into the *lag*-*2(q420)* background. Restoring *daf*-*16* function in neurons, but not in muscles, was sufficient to restore sleep bout increases in double mutant *daf*-*16*; *lag*-*2(q420)* animals (Fig. 3d). This result is consistent with previous work reporting a neuronal site of action for *daf*-*16* in homeostatic response to transient sleep deprivation [12].

**Fig. 4:**
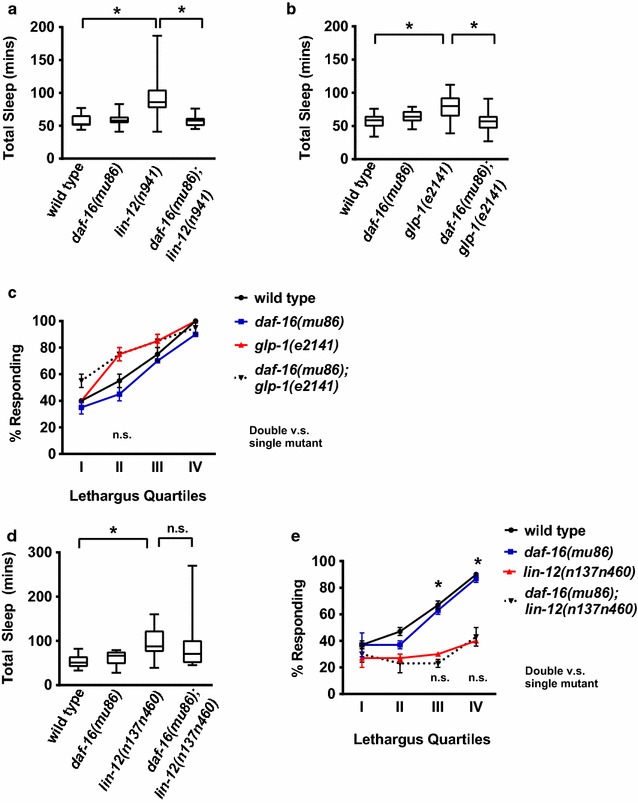
Decreased Notch signaling results in DAF-16 dependent increased sleep. **a** *lin*-*12* Notch receptor loss results in *daf*-*16*-dependent increased sleep. Animals reared at 25 °C and assayed at 22 °C. *lin*-*12(n941)* is a complete loss of function allele; *daf*-*16(mu86)* is a partial loss of function allele. Box shows two middle quartiles, horizontal line indicates mean, bars represent maximum/minimum; * denotes statistical significance by two-tailed student’s t-test with p ≤ 0.001 *versus* wild type. Wild type n = 8, *daf*-*16(mu86)* n = 10, *lin*-*12(n941)* n = 10, *daf*-*16(mu86);lin*-*12(n941)* n = 12. **b** Decreased *glp*-*1* Notch receptor function results in *daf*-*16*-dependent increased sleep. Parents shifted to 25 °C; F1 progeny assayed at 22 °C. *glp*-*1(e2141)* causes decreased function at higher temperatures; Statistical analysis as in panel A. *p ≤ 0.01 *versus* control. Wild type n = 14, *daf*-*16(mu86)* n = 13, *glp*-*1(e2141)* n = 14, *daf*-*16(mu86);glp*-*1(e2141)* n = 17. **c** Decreased *daf*-*16* function did not ameliorate arousal threshold defects in *glp*-*1* animals. Percent response reported for two independent trials; n = 20 animals for each data point with error bars for SEM. p = 0.11 for wild type versus *glp*-*1(e2141)* by t-test, but note that one way ANOVA suggests no statistical significance these genotypes or between *glp*-*1(e2141)* and *daf*-*16(mu86);glp1(e2141).* **d** Increased sleep quantity in *lin*-*12* gain of function animals is *daf*-*16*-independent. Animals reared at 15 °C, and L4 stage animals assayed at 22 °C for total time in sleep bouts. *lin*-*12(n137n460)* is a temperature-sensitive, gain of function allele with increased function at lower temperatures. Statistical analysis as in panel A;*p ≤ 0.001 versus wild type. Wild type n = 8, *daf*-*16(mu86)* n = 10, *lin*-*12(n137n460)* n = 10, *daf*-*16(mu86);lin*-*12(n137n460)* n = 12. **e** Increased arousal thresholds in *lin*-*12* gain of function animals are *daf*-*16*-independent. Statistical analysis as in panel C. Four independent trials, with n = 40 for each data point. p ≤ 0.001 *versus* wild type. No statistical significance found by student’s two-tailed t-test for *lin*-*12(n137n460) versus* double mutant

We found that DAF-16 also impacts sleep in other genotypes with reduced Notch signaling. We used two Notch receptor mutant alleles in this analysis: the null, complete loss of function allele *lin*-*12(n941)* and the temperature sensitive, loss of function allele *glp*-*1(e2141)*. Decreased *daf*-*16* function eliminated the increased sleep observed in both receptor loss of function backgrounds (Fig. 4a, b), indicating that *daf*-*16* function is required for increased sleep when Notch receptor function is decreased. The impact of *daf*-*16* on arousal during sleep bouts was tested in animals with decreased *glp*-*1* function. Previous work found that decreased *glp*-*1* function results in increased response to chemosensory stimulation during sleep [7]. At the restrictive temperature, we found arousal threshold defects in *glp*-*1(e2141)* animals approached statistical significance only at lethargus stage II (p = 0.11) based on pairwise student’s t-test, but not at other time points or using more stringent statistical tests. Arousal thresholds of double mutant *daf*-*16(mu86); glp*-*1(e2141)* animals at this time point were not different from those of *glp*-*1(e2141)* animals (Fig. 4c) by pairwise student’s t-test. We cautiously suggest that *daf*-*16* loss may not alter arousal thresholds in *glp*-*1* animals during sleep bouts, but *daf*-*16* activity is clearly required for sleep increases caused by diminished Notch signaling.

We considered two alternative hypotheses to explain these results: *daf*-*16* might be required downstream of Notch receptors for sleep increases or loss of *daf*-*16* might always decrease excessive sleep bout number. To distinguish between these hypotheses, we took advantage of a gain of function, cold-sensitive allele for the LIN-12 Notch receptor, *lin*-*12(n137n460)* [19]. Singh and colleagues found that *lin*-*12(n137n460)* animals have increased total sleep and increased response time only during sleep bouts [7]. We tested the impact of *daf*-*16* loss on sleep and arousal in *lin*-*12(n137n460)* animals. Double mutant animals retained the increased total time in sleep bouts and increased response times during sleep bouts of *lin*-*12(n137n460)* single mutant animals at later stages of lethargus (Fig. 4d, e). Yet, we found that decreasing *daf*-*16* function did not alter developmental defects or hypertonic stress resistance caused by decreased Notch function (Additional file 2: Table 1). Taken together, these results indicate that increased sleep caused by diminished Notch function during L4/A lethargus requires *daf*-*16* function, but that *daf*-*16* loss probably does not ameliorate arousal threshold defects seen in Notch receptor loss of function backgrounds. This is consistent with a requirement for DAF-16 FoxO for increased sleep bouts and a requirement for Notch signaling in establishing appropriate arousal thresholds during sleep bouts.

### Decreased JNK-1 signaling causes increased sleep that is DAF-16 FoxO-dependent

**Fig. 5:**
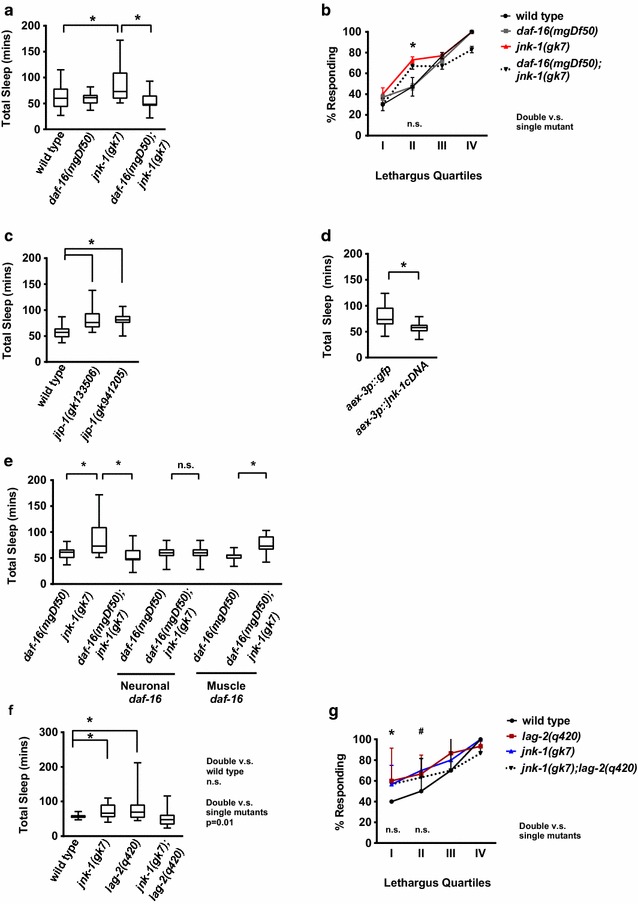
Loss of JNK-1 disrupts *C. elegans* sleep. **a** Loss of *jnk*-*1* results in *daf*-*16*-dependent increased sleep. *jnk*-*1(gk7)* and *daf*-*16(mgDf50)* are loss of function alleles. p ≤ 0.05 wild type *versus jnk*-*1(gk7)*, p ≤ 0.01 *jnk*-*1(gk7) versus daf*-*16(mgDf50);jnk*-*1(gk7)* assessed by student’s two tailed t-test. Wild type n = 13, *daf*-*16(mgDf50)* n = 12, *jnk*-*1(gk7)* n = 11, *daf*-*16(mgDf50);jnk*-*1(gk7)* n = 12. **b** *daf*-*16* loss does not ameliorate *jnk*-*1* arousal threshold defects during sleep bouts. Percent response for three trials, n = 30. p ≤ 0.05 wild type *versus jnk*-*1(gk7),* assessed by one way ANOVA followed by a Tukey post hoc-test; error bars respresent the SEM. **c** *jip*-*1* loss results in increased sleep. *jip*-*1(gk133506)* and *jip*-*1(gk941205)* are partial loss of function alleles. Analysis as in panel A; p ≤ 0.001*versus* wild type. Wild type n = 13, *jip*-*1(gk133506)* n = 12, *jip*-*1(gk941205)* n = 10. **d** *jnk*-*1* is required in neurons to regulate sleep. Transgenic *jnk*-*1(gk7)* animals with aex-*3* promoter expressing either GFP or *jnk*-*1* cDNA expression were tested. Significance as in panel A. *aex*-*3p::gfp;jnk*-*1(gk7)* n = 8 and *aex*-*3p::jnk*-*1cDNA;jnk*-*1(gk7)* n = 13. **e** *daf*-*16* function is required in muscles for increased sleep in *jnk*-*1* animals. DAF-16 was expressed under the control of a neuron or muscle-specific promoter in *daf*-*16(mgDf50); jnk*-*1(gk7)* animals. p ≤ 0.01 *jnk*-*1(gk7)* to *daf*-*16(mgDf50);jnk*-*1(gk7),* p ≤ 0.001 DAF-16 muscle rescue *versus* control. *daf*-*16(mgD50)* n = 12, *jnk*-*1(gk7)* n = 11, *daf*-*16(mgDf50);jnk*-*1(gk7)* n = 12, *Punc*-*119:daf*-*16(mgDf50)* n = 14, *Punc*-*119:daf*-*16(mgDf50);jnk*-*1(gk7*) n = 12, *Pmyo*-*3:daf*-*16(mgDf50)* n = 10, *Pmyo*-*3:daf*-*16(mgDf50);jnk*-*1(gk7)* n = 12. Significance as in panel A. **f** *jnk*-*1* and *lag*-*2* both contribute to regulate sleep quantity. Analysis as in panel A; p ≤ 0.03 wild type *versus jnk*-*1(gk7),* p ≤ 0.05 wild type *versus lag*-*2(q420),* p ≤ 0.01 *jnk*-*1(gk7);lag*-*2(q420)* *versus jnk*-*1(gk7)* and *lag*-*2(q420).* Wild type n = 12, *lag*-*2(q420)* n = 13, *jnk*-*1(gk7*) n = 11, *jnk*-*1(gk7);lag*-*2(q420)* n = 20. Significance as in panel A. **g** Loss of both *jnk*-*1* and lag-2 further decreases arousal thresholds during sleep bouts. Analysis as in B, three independent trials, n = 30 animals

In our experience, it is uncommon to observe decreased arousal thresholds during sleep bouts in genotypes with increased sleep during *C. elegans* lethargus. In almost all genotypes, decreased arousal thresholds during sleep bouts are seen in mutant genotypes with decreased total sleep, and vice versa, as we have discussed previously [8, 25]. To our knowledge, the only other discordant *C. elegans* gene is *jnk*-*1*, whose loss causes increased total sleep bout quantity with decreased response during sleep bouts. *jnk*-*1* encodes an orthologue of the mammalian c-Jun N terminal kinase protein [36]. Is increased sleep in animals lacking *jnk*-*1* also dependent on *daf*-*16* function? When food was plenteous, *jnk*-*1(gk7)* animals, which completely lack *jnk*-*1* function, had increased total time in sleep bouts (Fig. 5a) as previously reported [7]. Loss of FoxO function in *daf*-*16(mgDf50) jnk*-*1(gk7)* double mutant animals restored wild type levels of total time in sleep bouts and normal lethargus duration (Fig. 5a, Additional file 5: Raw Data File). Yet, *daf*-*16(mgDf50) jnk*-*1(gk7)* animals retained the decreased arousal thresholds during sleep bouts of early lethargus that were observed in *jnk*-*1(gk7)* animals (Fig. 5b). Therefore, similar to results seen with *lag*-*2,* we find that decreased JNK-1 signaling leads to increased sleep with decreased arousal thresholds.

To confirm the impact of the JNK pathway on *C. elegans* sleep, we examined the role of another pathway component. In mammals, JNK-interacting protein 1 (JIP-1, also known as MAPK8IP1) scaffolds JNK-1 interactions with target proteins, leading to increased activity and specificity. JNK-1 functions upstream of the FoxO transcription factor, which is activated by increased JNK signaling [37, 38]. To determine if loss of the *C. elegans* JNK-1 orthologue *jip*-*1* caused sleep defects, we obtained two *jip*-*1* partial loss-of-function alleles. We found that decreased *jip*-*1* function resulted in increased total time in sleep bouts, when compared to wild type animals (Fig. 5c). This is consistent with results from *jnk*-*1* loss of function animals and confirms that loss of function in this pathway increases lethargus sleep bouts.

To determine where *jnk*-*1* function was required, we used tissue-specific knockdown by RNAi. Feeding *C. elegans* bacteria that express double-stranded RNA for a *C. elegans* gene is effective to knock down target mRNAs in most somatic tissues. However, neurons are among the few tissues that are refractory to this approach. Expression of the double-stranded RNA channel SID-1 in neurons renders them more sensitive to RNAi by feeding [16]. Previous work demonstrated that RNAi knockdown of *jnk*-*1* in animals expressing neuronal SID-1 resulted in increased total time in sleep bouts and recapitulated the defects seen in *jnk*-*1(gk7)* animals [8]. We suspected that *jnk*-*1* might function in neurons. We tested this hypothesis by generating transgenic animals expressing *jnk*-*1* cDNA under a pan-neuronal promoter into *jnk*-*1(gk7)* animals. We found that restoring *jnk*-*1* expression in neurons was sufficient to restore sleep bouts to wild type levels (Fig. 5d). We also tested this hypothesis using animals that do not express ectopic SID-1 in neurons. In these animals, *jnk*-*1* RNAi knockdown had no impact on total sleep bout number or L4/A lethargus duration (Additional file 8: Fig. 5, Additional file 5: Raw Data File). Confirmation of RNAi knockdown was not undertaken and it is possible that *jnk*-*1* levels were not decreased and we cannot rule out a role for JNK-1 outside the nervous system. However, these independent rescue and RNAi results suggest that loss of *jnk*-*1* function in neurons likely leads to increased sleep bouts and decreased response times during L4/A lethargus sleep bouts.

To determine where *daf*-*16* function is required for increased sleep in animals lacking *jnk*-*1*, we generated *jnk*-*1(gk7)* animals carrying transgenes that drive *daf*-*16* expression under the control of a pan-neuronal promoter or a muscle-specific promoter. Restoring *daf*-*16* function in muscles, but not in neurons, was sufficient to restore increased sleep bout numbers in *jnk*-*1(null)* mutant animals (Fig. 5e). A requirement for *daf*-*16* in the body wall muscle is consistent with previous work showing that DAF-16 function is necessary for compensatory sleep changes after mechanical-stress induced sleep bout deprivation or in animals with decreased insulin receptor signaling [11, 39]. We and others have noted that the site of *daf*-*16* action varies depending on the type sleep perturbation [11, 12]. It is possible that activation of DAF-16 FoxO in any one of several key tissues is sufficient to induce sleep changes, reminiscent of the multifocal sites of DAF-16 action in aging and metabolism [40, 41].

### LAG-2 and JNK-1 may function in different genetic pathways

Previous work identified a complex and antagonistic relationship between the Notch and JNK-1 signaling pathways, based on binding to JIP-1 [42]. However, in *C. elegans* we found that decreased signaling in either Notch or JNK pathways increased time in L4/A lethargus sleep bouts and decreased arousal thresholds during sleep bouts. This makes it less likely that JIP-1 regulates cross talk between JNK-1 and Notch pathways in this context.

To examine the relationship between JNK-1 and Notch pathways, we tested the consequences of simultaneously perturbing both *jnk*-*1* and *lag*-*2*. If these two genes function in exactly the same pathway for sleep and/or arousal, then the double mutant should have defects identical to *jnk*-*1* or *lag*-*2(q420)* animals. But, complete loss of *jnk*-*1* function in *lag*-*2(q420)* double mutant animals resulted in restoration of normal sleep bout quantity, in contrast to the increased sleep seen in either single mutant (Fig. 5f). When we examined arousal thresholds, *jnk*-*1(gk7); lag*-*2(q420)* double mutant animals resembled single mutant animals; arousal thresholds during sleep bouts observed in either *lag*-*2(q420)* or *jnk*-*1(gk7)* single mutants in the early stages of lethargus were not different than double mutant animals in in the first half of lethargus (Fig. 5g). Because the double mutants have normal total sleep (unlike single mutants), yet double mutants had similar arousal threshold defects during sleep bouts, we suggest that the interactions between these pathways for sleep are complex and cannot be reduced to linear pathways. It is possible that these genes function in a convergent pathway for arousal thresholds, but their relationship is more complex in sleep.

### *C. elegans* lacking DAF-16 can survive L4/A lethargus without sleep bouts

**Fig. 6:**
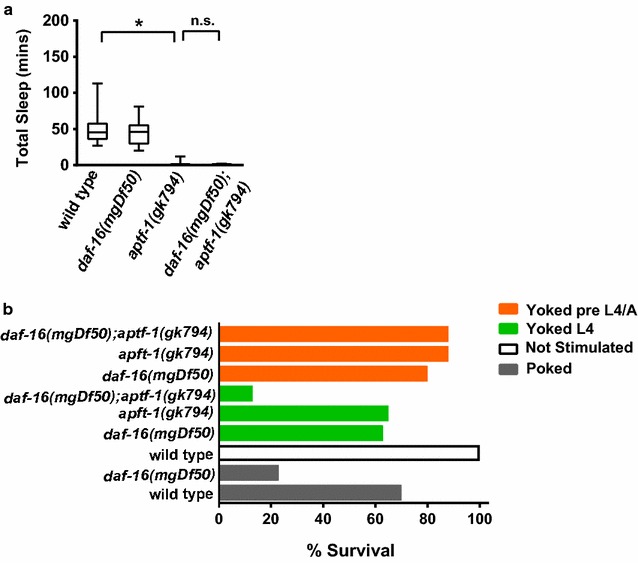
Mechanical stress with lack of sleep bouts results in increased death. **a** Loss of *daf*-*16* does not alter sleep quantity in animals lacking *aptf*-*1.* Animals were reared at 25 °C and assayed at 22 °C. Total time in sleep bouts reported in minutes. Box shows two middle quartiles, horizontal line indicates mean, bars represent maximum/minimum. Statistical significance by student’s two tailed t-test noted as * for p ≤ 0.001 versus wild type. Wild type n = 10, *daf*-*16(mgDf50)* n = 9, *aptf*-*1(gk794)* n = 10, *daf*-*16(mgDf50);aptf*-*1(gk794)* n = 11. **b** Mechanical stress, in combination with lack of sleep bouts, results in death. *daf*-*16 (mgDf50), aptf*-*1(gk794)*, and *daf*-*16(mgDf50);aptf*-*1(gk794)* animals were selected at the beginning of the L4 lethargus. To deprive animals of sleep, animals were physically stimulated in the tail region using a platinum wire upon entering a L4/A lethargus sleep bout, as marked by cessation of locomotion and pharyngeal pumping. These animals are referred to as “poked”. Yoked animals were physically stimulated with the same force whenever poked animals were prodded, regardless if in sleep or motion bout. Stimulation continued over a roughly 40 min time interval and ended when poked animals no longer responded to stimulus. Control animals were not stimulated. Survival was determined 24 h after stimulation based on ability to move and/or feed. Animals: n = 23 for all genotypes. p ≤ 0.0001 (with 2 degrees of freedom) as assessed by chi square test

Decreased sleep or sleep deprivation is deleterious. Enforced prolonged sleep deprivation in mammals and *Drosophila* by mechanical perturbation leads to death [9, 43]. Similarly, enforced sleep bout deprivation during the first part of *C. elegans* L4/A lethargus leads to decreased survival in animals lacking DAF-16 FoxO [11]. When undertaking studies described herein, there was concern that poor quality sleep bouts might lead to death in genotypes we examined. Yet, neither diminished Notch signaling nor loss of JNK1 signaling caused death during or immediately after L4/A lethargus, even in animals lacking *daf*-*16*. It seemed possible that residual sleep bouts in double mutant animals might be sufficient for survival, despite being of poor quality. Turek *et al.* reported that reduced function in APTF-1, a *C. elegans* AP2 transcription factor, results in a complete lack of motionless sleep bouts during lethargus, which we confirmed (Fig. 6a). Animals lacking *aptf*-*1* display common features of diapause and lethargus (cuticle shedding, cessation of pharyngeal pumping, altered neuronal excitability), with a selective defect in sleep bouts. Specifically, these mutant animals are continuously active during lethargus with wake-like locomotion patterns and rapid response to sensory stimulation during lethargus [44], which we also confirmed. All animals lacking *aptf*-*1* survive lethargus (Additional file 3: Table 2), as previously reported [44]. This seemed at odds with the requirement for sleep bouts implicit in Driver *et al.* as *daf*-*16*-dependent compensatory sleep bouts are required for survival after mechanical sleep deprivation in several different experimental paradigms. It seemed plausible that *aptf*-*1* animals might survive because *daf*-*16*-dependent compensatory responses lead to restoration of sleep bouts. Therefore, we examined *daf*-*16(mgDf50);aptf*-*1(gk794)* animals. No sleep bouts were detected in these animals (Fig. 6a) and to our surprise all animals survived L4/A lethargus (Additional file 3: Table 2). It was possible that motion bouts and sleep bouts are equally restorative in *aptf*-*1* animals or possible that sleep bouts are more restorative, but required only under specific conditions.

In animal studies, enforced sleep deprivation is generally induced using strong mechanical stimulation over extended time periods [11, 45]. Perhaps, forced motion alone is not sufficient to induce death and stress contributes? We considered alternative models in which the two insults-forced motion and mechanical stress, might act independently or synergistically, leading to death.

To determine if mechanical stimulation acts in combination with sleep bout loss to induce death, we developed a modified version of the previous described mechanical stress sleep deprivation approach [11]. Strong mechanical stimulation was used to disturb *C. elegans* sleep bouts during the first 40 min of the L4/A lethargus. In one group, called “poked”, sleep bouts were prevented by prodding animals with a wire pick near the tail whenever locomotion stopped. In the second group, called “yoked”, stage-matched animals were prodded with similar force and frequency, regardless of whether they were in a sleep bout or a motion bout. After 24 h, all animals were scored for survival. Unperturbed animals always survived for all genotypes tested. We note that yoked animals act as mechanical stress controls; prodding yoked animals disturbs only 20% of their sleep bouts.

First, we confirmed previous results showing that DAF-16 FoxO is protective in this paradigm [11]. All yoked wild type animals survived, but 30% of wild type animals poked during sleep bouts died (Fig. 6b, 70% surviving). This is consistent with previous work, but the death rate here is higher. We attribute the difference to how animals were prodded. In the previous study animals were prodded in liquid [11], but here, animals were prodded on the surface of an agar plate, which may be more traumatic. Nevertheless, loss of *daf*-*16* was deleterious. Almost 80% of *daf*-*16(mgDf50)* animals died when poked only during sleep bouts and almost 40% of yoked *daf*-*16* animals died (Fig. 6b). Our results confirm that mechanical sleep deprivation which perturbs sleep bouts is detrimental to *C. elegans* and that loss of DAF-16 FoxO decreases survival. Yet, these results are not sufficient to disentangle the relative deleterious impacts of mechanical damage *versus* forced motion.

If mechanical stress only induces sleep deprivation and is not otherwise damaging, then *aptf*-*1* animals should survive mechanical stimulation during L4/A lethargus as well as wild type animals. Yet, we found that mechanical stimulation during L4/A lethargus was more deleterious to animals lacking *aptf*-*1*, compared to wild type animals. No yoked wild type animals died after mechanical stimulation during L4/A lethargus, but 35% of yoked *aptf*-*1(gk794)* animals died. (Poked results cannot be obtained for *aptf*-*1* mutant animals as they have no sleep bouts.) These results suggested that sleep bouts or compensatory sleep might help wild type animals survive damaging mechanical stimulation. In addition, these results suggest that loss of sleep bouts may be deleterious to *aptf*-*1* animals under some conditions.

To assess if sleep bouts contribute to surviving mechanical stress, we used two different experimental strategies. In the first, sleep bouts were eliminated during and after lethargus by testing yoked *daf*-*16(mgDf50);aptf*-*1(gk794)* animals. Over 80% of these animals died, consistent with a role for *daf*-*16* in mechanical stress resistance, which may be independent of the established role for *daf*-*16* in compensatory sleep. In the second experimental strategy, mechanical stress and sleep bout disruption were uncoupled temporally: animals were subjected to the yoked prodding during the L4 larval stage, prior to lethargus sleep onset. Under these conditions; there was not a significant difference between yoked *daf*-*16(mgDf50)* animals, yoked *aptf*-*1(gk794)* animals, or yoked double mutant *daf*-*16(mgDf50)*;*aptf*-*1(gk794)* animals (Fig. 6b). In each genotype, 15–20% of animals died during or after the subsequent L4/A lethargus. These results suggest that mechanical stress during L4/A lethargus is uniquely stressful and that loss of sleep bouts at this stage may sensitize animals to stress. Combined, these studies confirm that lethargus sleep bouts are not required for *C. elegans* survival under standard culture conditions. And, they suggest that normal sleep bouts and FoxO may work in parallel to help animals survive mechanical stress. Also, they suggest that motion bouts are not as restorative as sleep bouts, although other restorative functions of the lethargus developmental stage may be preserved in *aptf*-*1* mutants.

## Discussion

Here, we examine the relationship between stress-responsive signaling pathways and sleep, using genetic and behavioral approaches in *C. elegans*. We define a role for the Notch ligand LAG-2 and the JNK pathway in *C. elegans* sleep bouts and we examine the requirement for DAF-16 FoxO when sleep bouts are perturbed. We find that DAF-16 FoxO function is required for regulating sleep bout quantity in specific scenarios and we find that *C. elegans* sleep bouts help animals survive mechanical stress during lethargus. These results may have implications for sleep deprivation studies in other animal species.

Previous work reported that sleep-related behaviors during the transition to *C. elegans* adulthood are sensitive to changes in Notch receptor signaling [7, 8]. *C. elegans* Notch receptors and ligands work synergistically to regulate sleep bout quantity. Increased Notch pathway signaling drives ectopic sleep bouts in adults, increases sleep bout number during lethargus, and increases arousal thresholds during sleep bouts. Thresholds for sensory response or locomotion during lethargus motion bouts are not affected. It remains unclear why both increased and decreased Notch signaling lead to increased sleep. It was suggested that increased Notch signaling leads to increased sleep bout number and deeper sleep during these bouts [7]. And, it was suggested that decreased Notch signaling results in more complex changes. Specifically, loss of one receptor or one ligand results in increased sleep bout quantity with decreased arousal thresholds, while loss of more than one ligand results decreased sleep bout number and further decreased arousal thresholds. Again, thresholds for sensory response or locomotion during motion bouts were unaffected. Combined, these results suggested the hypothesis that decreased Notch signaling results in shallower sleep during sleep bouts, but when residual Notch is sufficient, animals can engage homeostatic pathways to compensate and increase sleep bout number. But, given the paucity of our knowledge about sleep homeostatic mechanisms, this hypothesis could not be further examined at that time. Subsequent studies revealed that DAF-16 FoxO function is required for compensatory changes when *C. elegans* sleep is mechanically perturbed [11, 12] which allowed us to further test this hypothesis in this study.

To define more carefully the changes in sleep bout quantity and arousal thresholds caused by defects in the Notch pathway, we focused on the Notch ligand LAG-2 and the JNK pathway. We took advantage of a previously described, temperature-sensitive *lag*-*2* allele that allowed us to extend previous work and confirm that manipulating Notch signaling specifically altered sleep bout number and arousal thresholds during sleep bouts. The compensatory sleep hypothesis predicted that loss of DAF-16 function would decrease sleep bout number, but not alter arousal thresholds during sleep. As predicted, we found that DAF-16 function was required for the increased number of sleep bouts in *lag*-*2* mutant animals, but DAF-16 loss had no impact on arousal thresholds. Sensory response during motion bouts was unaffected at restrictive temperatures and arousal thresholds during sleep bouts in the last half of lethargus are also unaffected. A similar constellation of behavioral defects was confirmed here in animals with defects in JNK pathway signaling: decreased arousal thresholds during sleep bouts and increased sleep bout number. Again, loss of DAF-16 function ameliorated sleep bout quantity defects without impacting arousal thresholds during sleep bouts. These results are consistent with the hypothesis tested here; decreased Notch or JNK signaling leads to poor quality, non-restorative sleep bouts, which engages compensatory mechanisms that lead to increased sleep.

It seems unlikely that decreased *lag*-*2* function globally depresses locomotion activity or generically makes animals hypersensitive to stimulation. Animals with decreased *lag*-*2* function (1) have normal sensory response during motion bouts or in adult animals, (2) have normal or increased activity levels in adult animals [7] (3) have grossly normal locomotion/activity in L4 motion bouts or after 1 h of adulthood, and (4) have normal overall timing of development/diapause, based on vulval eversion. Therefore, altered *lag*-*2* activity does not change basal activity or arousal.

These results are consistent with the hypothesis that in animals with decreased Notch or JNK signaling and decreased arousal thresholds engage compensatory DAF-16 sleep mechanisms. However, it is also possible that changes in arousal thresholds are independent of changes in sleep. In this alternative hypothesis, Notch and JNK pathways regulate sleep via a DAF-16-dependent pathway, but regulate arousal thresholds via a non-overlapping DAF-16-independent pathway. Future studies will be required to identify the mechanisms by which Notch and JNK regulate sleep and arousal, to rule out one of these two hypotheses.

The cellular and molecular mechanisms that alter arousal and sleep in animals with decreased Notch signaling are unclear. Decreased Notch signaling has been implicated previously in stress response. When *C. elegans* are exposed to excessive hypertonic stress (e.g. high external NaCl), this leads to decreased secretion of the Notch co-ligand OSM-11. Ultimately, this results in global decreases in somatic Notch signaling [46]. The consequences are numerous and include increased immune response, as well as increased internal glycerol levels, which restores osmotic balance [46–48]. Modestly decreased Notch signaling may engage these stress response pathways, resulting in changes in arousal thresholds and/or sleep bouts. It should be noted that increased arousal is not global or dramatic, as sensory response thresholds do not change during motion bouts or outside lethargus. Indeed, modestly decreased Notch signaling generally decreases response to noxious stimulation, such as octanol. JNK pathway signaling is involved in stress response and a similar constellation of sleep defects is seen when signaling in the JNK-1 pathway is perturbed. It is tempting to draw parallels across species and suggest that chronic changes in stress pathway signaling lead to arousal threshold changes in sleep bouts by common mechanisms. However, until the mechanisms driving these arousal threshold changes are fully delineated, these parallels cannot be established.

We find that DAF-16 FoxO is required for sleep bout increases in animals with decreased *lag*-*2* function. However, there is no evidence that decreased Notch signaling directly activates DAF-16 FoxO. DAF-16 loss had no impact on the aberrantly low response times during sleep in *lag*-*2* animals. Furthermore, in otherwise normal animals, LAG-2 loss did not impact adaptation to hypertonic stress response. This is consistent with decreased Notch signaling (specifically, poor quality sleep) being sufficient to induce increased sleep via a DAF-16 dependent pathway. The molecular or cellular pathways by which DAF-16 FoxO drives increased sleep remain unclear. Animals with decreased Notch or JNK pathway signaling are a tractable genetic background that could be used to dissect DAF-16 dependent pathways leading to increased sleep.

Activation of DAF-16 or JNK-1 is important in initiating stress response across species [49]. DAF-16 is an important regulator of diverse stress responses [50, 51] and DAF-16 loss in various tissues impacts longevity and stress resistance [52–55]. DAF-16 signaling is highly sensitive to food availability; we undertook analyses here in the presence of plentiful food. When food is not plentiful, there is a dramatic change in sleep bouts during the L4/A lethargus. Additional studies will be required to define how genotype interacts with dietary stress in the context of sleep. We consider it unlikely that *daf*-*16* loss of function alone causes hypersensitivity to damage based on two observations. First, animals with decreased *daf*-*16* function were not more likely to die after mechanical perturbation, as mechanical perturbation of yoked L4/A or yoked pre-lethargus *daf*-*16(mgDf50)* animals did not cause more death than in wild type or *aptf*-*1* loss of function animals (Fig. 6b). Second, manipulating *daf*-*16* animals for microfluidic chamber assays did not decrease their basal activity levels. The simplest conclusion that can be drawn from results presented here and elsewhere is that *daf*-*16* is required for response to decreased and/or disturbed sleep bouts.

**Fig. 7:**
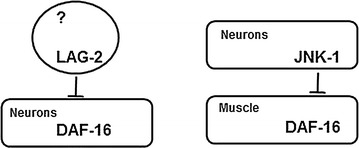
DAF-16 can act in multiple tissues to regulate sleep. Summary figure: decreased function of the Notch ligand *lag*-*2* or loss of the stress response kinase *jnk*-*1* increased total time in sleep bouts, dependent on DAF-16/FOXO function. Sleep is dependent on DAF-16 function in neurons of animals with decreased *lag*-*2* function. The neurons and/or tissues where Notch DSL ligand LAG-2 functions in sleep regulation have not been identified. Previous work demonstrated that JNK-1 function is required in neurons for sleep bout quantity and sleep bout arousal thresholds. However, increased sleep in *jnk*-*1* loss of function animals is dependent on DAF-16 function in muscles. We propose communication between muscles and neurons is required for DAF-16 dependent increased sleep in *C. elegans* L4/A lethargus and that DAF-16 can act in a non-cell-autonomous manner to regulate compensatory sleep bouts in *jnk*-*1* mutant animals

JNK-1 can activate DAF-16, which promotes nuclear entry and activation of DAF-16 target genes. However, we found that DAF-16 function is required for increased sleep in animals lacking *jnk*-*1*, which argues against JNK-1 directly activating DAF-16 in this context. Moreover, we found that JNK-1 and DAF-16 may function in different tissues. JNK-1 likely functions in neurons to regulate sleep bouts [8]. DAF-16 expression in muscles restored sleep in *daf*-*16(null);jnk*-*1(null)* double mutant animals to levels seen in *jnk*-*1(null)* animals (Fig. 5). This is consistent with previous studies demonstrating that DAF-16 expression in muscles is sufficient for homeostatic sleep responses after mechanical stimulation [11]. Therefore, factors other than JNK-1 likely act directly upstream of DAF-16 to regulate sleep bouts. Additionally, results presented here suggest crosstalk between neurons and muscles in the regulation of sleep. Inadequate sleep may result in non-autonomous stress signaling that would activate DAF-16, resulting in increased sleep (Fig. 7). Loss of *daf*-*16* hypersensitizes animals to mechanical stress, presumably because they are unable to compensate by increasing arousal thresholds and/or sleep bout quantity. The fact that *daf*-*16* is involved in these sleep alterations suggests that a stress response pathway is activated in response to poor quality or lost sleep.

Sleep bouts are sensitive to changes in Notch, JNK-1, and FoxO signaling, as well as mechanical stress. We point out that it is unclear if motion bouts during lethargus are a sleep-like state. There are pervasive changes during L4/A lethargus that can be observed even during motion bouts. For example, lethargus animals have altered response to aldicarb, an inhibitor of acetylcholinesterase [56]. Optical imaging studies suggest decreased sensory neuron responsiveness during both sleep and motion bouts of lethargus. Also, pharyngeal muscles intrinsic excitability is dramatically decreased during the entirety of lethargus, during both sleep and motion bouts. This contributes to the lack of feeding during lethargus [6]. Combined, these results suggest that cellular excitability shifts during lethargus, with consequences for both sleep and motion bouts. Yet, several lines of evidence suggest that sleep bouts are distinct from motion bouts. Arousal threshold changes and lack of locomotion characteristic of sleep are seen only in sleep bouts. Results here and previous work suggest that loss of sleep bouts is deleterious when combined with mechanical stress. Combined, these results suggest that sleep bouts are distinct and uniquely restorative; motion bouts are not as restorative as sleep bouts.

We find that LAG-2 and JNK-1 may act in the same pathway for arousal thresholds, but may have a more complex relationship in their regulation of sleep quantity. Total sleep in double mutant *jnk*-*1(null);lag2(tslf)* animals was equal to that of control animals, despite the fact that either single mutant had increased sleep. The synthetic suppression of sleep defects in the *jnk*-*1 lag*-*2* double mutant suggests that- at least in part, *lag*-*2* and *jnk*-*1* act in distinct pathways to disrupt sleep. Another line of evidence indicating that Notch and JNK signaling impact sleep through distinct pathways is that different tissues require DAF-16 function when these two pathways are perturbed. In animals with decreased *lag*-*2* function, DAF-16 expression is required in neurons, but not muscles, for sleep increases. Yet, DAF-16 expression in muscles, but not neurons, is required for sleep increases in animals lacking *jnk*-*1* (Figs. 3, 4). It seems likely that Notch and JNK act in complex, but convergent, pathways in different tissues that impact sleep.

Prolonged periods of sleep deprivation result in decreased intellectual performance [57, 58], and skin lesions [9, 59, 60]. Usually, strong mechanical stimulation is used to induce sleep deprivation. We and others have found that lack of sleep bouts due to genetic manipulation in *C. elegans* is not sufficient to induce death, but we report that loss of sleep bouts increases vulnerability to mechanical perturbation. Loss of the APTF-1 virtually eliminates sleep bouts, but does not cause lethality [44]. Our results demonstrate that motion bouts in *aptf*-*1* mutant animals are not as restorative as motionless sleep bouts, although lethargus sleep bouts are non-essential. To our surprise, animals lacking both *aptf*-*1* and *daf*-*16* were viable (Additional file 3: Table 2). This indicates that *C. elegans* sleep bouts are not required for survival under optimal growth conditions. To determine if loss of sleep bouts contributes to stress resistance in *C. elegans*, we examine the interaction of reduced sleep bout number and mechanical stress. We found that mild mechanical perturbation of either *daf*-*16* or *aptf*-*1* (yoked) animals sometimes resulted in death, while animals lacking both genes had higher death rates. This argues that DAF-16 also contributes to survival independently of either APTF-1 or reduced sleep bouts.

To look for contributions of APTF-1 to mechanical stress survival, independent of the APTF-1 role in sleep bouts, we examined the impact of mechanical stress during the last larval stage, prior to lethargus. We found that mechanical stress during the L4 larval stage was not as detrimental; death due to mild mechanical stress during the L4 larval stages was dramatically less in all genotypes, when compared to mechanical stress occurring during lethargus. This suggests that sleep bouts, and not *aptf*-*1* function *per se*, are important for surviving mechanical stress. Moreover, this suggests that mechanical stress is most harmful when sleep bouts are disturbed. It is not clear why mammals or *C. elegans* die after prolonged sleep deprivation.

Allostatic load refers to the physiological cost to the body over time caused by adaptation to overall stress [61, 62]. It has been proposed that an increase in allostatic load, due to persistent lack of sleep, makes mammals more sensitive to additional stress and more likely to develop disease [62]. Analogously, we hypothesize that the sleep bout-deprived animals lacking DAF-16 FoxO have an increased allostatic load that makes them more susceptible to additional mechanical stress. We suspect that previous studies using other species that used mechanical stimulation to induce sleep deprivation likely also introduced significant mechanical stress, which may have contributed to death ascribed to sleep deprivation alone. Other techniques have been used to experimentally deprive animals of sleep, including light-induced stimulation, motion, and pharmacological agents [63–65]. These methods decrease, but do not prevent sleep; varying amounts of rebound sleep are observed. Interestingly, death has not been observed when these less damaging stimuli are used as sleep deprivation methods. This is consistent with our conclusion in *C. elegans;* strong mechanical stress may be a confounding factor in previous studies suggesting that lack of fully restorative sleep is sufficient to cause rapid death.

Disentangling the deleterious effects of sleep deprivation from the deleterious effects of mechanical stress will be important in future studies of sleep deprivation and sleep homeostasis. Overall, this work demonstrates that Notch signaling and JNK-1 signaling regulate arousal thresholds and sleep bout quantity. DAF-16 FoxO function is required for the increased sleep of animals with decreased Notch or JNK signaling. But, arousal threshold defects are not DAF-16 dependent. These results suggest that either poor sleep quality leads to DAF-16-dependent increases in sleep quantity- or that these signaling pathways regulate sleep and arousal via different mechanisms. In addition, this study suggests that unidentified pathways act in parallel to DAF-16 to help sleep bout-deprived animals survive mechanical stress. Given the deep conservation of Notch signaling, JNK-1 signaling, and DAF-16/FoxO transcription factors, these general mechanisms likely underlie sleep regulation and response to sleep deprivation across animal species.

## Conclusions

Sleep is a conserved behavior; however the mechanisms underlying homeostatic response to insufficient sleep are poorly understood. Here, we demonstrated that decreased function of the Notch ligand encoded by *lag*-*2* or loss of genes in the JNK/*jnk*-*1* signaling pathway, resulted in shallow sleep depth with increased sleep bout duration. This combination is consistent with a model in which homeostatic mechanisms drive increased sleep quantity to compensate for decreased sleep quality. Changes in sleep quantity reported here were dependent on *daf*-*16*, a FoxO transcription factor.

We also examined the consequences of sleep deprivation in *C. elegans* and find that lack of developmentally-timed sleep is not sufficient to induce death. However, we find that sleep is required to overcome concomitant stress caused by mechanical perturbation. Studies of sleep deprivation in other animals frequently use mechanical stress to prevent sleep. Our work here suggests that mechanical damage may contribute to death caused by sleep deprivation.

## Additional files

**Additional file 1:** Food concentration alters the quantity of L4/A lethargus motionless sleep bouts. Left: the total amount in sleep bouts (sum of durations of all quiescent bouts) of undisrupted wild-type animals, raised and assayed at 25 °C. Right: the total time in sleep bouts of animals repeatedly exposed to mechanical vibrations (10 s of 1 kHz vibrations every 10 min). Excess motion caused by the mechanical stimulus was compensated for and the resulting total time in sleep bouts increased with food concentration, exhibiting a similar trend to the case of undisrupted animals. Horizontal lines, inner boxes, and outer boxes depict means, standard errors of the mean, and standard deviations, respectively. Sample sizes are noted in parentheses and double asterisks denote significant differences (p < 0.01).

**Additional file 2:** *daf-16* is not required for hypertonic stress resistance in *osm-7; osm-11* animals. Hypertonic stress resistance was examined in young adult animals moved to 500mM NaCl NGM plates for 10 min. *osm-7(tm2256) and osm-11(rt142)* are complete loss of function alleles for Notch DOS family co-ligands*. daf-16(mu86)* is a partial loss of function allele. Loss of Notch DOS co-ligands results in resistance to hypertonic stress, based on inability to move, spontaneously or upon prodding, after 10 min on 500mM NaCl NGM plates. Partial loss of *daf-16* function does not alter hypertonic resistance in these animals. *n* = 40 animals for all genotypes.

**Additional file 3:** Simultaneous loss of *daf-16* and *aptf-1* does not decrease survival. Survival after L4/A lethargus is reported as percentage of animals failing to shed larval cuticle, undergo vulval eversion, and/or reach adult stage with normal locomotion and response to touch. The number of dead animals (animals not moving or feeding after prodding) was scored at 6 and 24 h after the start of L4/A lethargus. *n* = 75 animals for all genotypes.

**Additional file 4:** Notch ligands and sleep during L4/A lethargus. A) Increased time in lethargus results in increased sleep in animals with decreased *lag-2* function. Detailed breakdown of sleep metrics for wild type and *lag-2(q420)* animals at 25.5 °C. Average sleep bout duration in seconds and average number of sleep bouts is reported per hour. Statistical significance assessed by student’s two tailed t-test; * denotes p < 0.005 for wild type *versus lag-2(q420)*. All results available in supplemental raw data file for this and other panels. B) Partial loss of *lag-2* function results in increased lethargus duration. *lag-2(q420)* is a temperature-sensitive, loss of function allele with decreased function at higher temperatures. Lethargus duration was assessed during L4/A lethargus, wild type or *lag-2(q420)* animals were shifted from 15 °C to the restrictive temperature of 25.5 °C. Total lethargus duration during L4/A lethargus were examined in F1 progeny at 25.5 °C. Lethargus duration is reported in hours; error bars indicate SEM. Note that lethargus is determined by presence/absence of motionless sleep bouts, not by morphological/developmental criteria. Statistical significance was assessed by student’s two tailed t-test; * denotes p < 0.02. At 15 °C, wild type n = 13, *lag-2(q420)* n = 13. At 25.5 °C wild type n = 13, *lag-2(q420)* n = 14. C) Loss of *dsl-1* does not alter sleep. Progeny of animals were reared at 25 °C were assayed at 22 °C for total time in sleep bouts during L4/A lethargus. *dsl-1(ok810)* is a complete loss of function allele. Results presented as a box plot. Wild type n = 17, *dsl-1(ok810)* n = 16. No difference between genotypes based on student’s two tailed t-test.

**Additional file 5:** Raw Data File.

**Additional file 6:** Decreased *lag-2* function does not slow vulval development. The progeny of wild type and *lag-2(q420)* animals raised at 25.5 °C were selected at the L4 stage, prior to lethargus entry. Vulval eversion was scored after 3 h; the percentage of animals completing vulval eversion was recorded. Significance was assessed by student’s two-tailed t-test p value < 0.5; error bars represents SEM from 3 trials. Total number of animals: wild type n = 45 and *lag-2(q420)* n = 42.

**Additional file 7:** Increased *lag-2* expression in adults induces anachronistic sleep bouts. Animals carrying *hsp::empty*, *hsp::lag-2 cDNA* transgenes, or wild type animals were heat shocked for 1.5 h at 34 °C. After heat shock, animals were allowed to recover at 20 °C for an additional 1 h to recover from stress-induced quiescence (shown in first set of columns). Sleep was scored for all genotypes within 15 min, based on the absence of feeding and movement. Inappropriate sleep in adult animals expressing *hsp::lag-2cDNA* transgene was reversible, disappearing by 2 h post-heat shock. For all genotypes, n = 40 animals; error bars represent the SEM from 2 independent trials.

**Additional file 8:** *jnk-1* RNAi knockdown does not alter sleep bout quantity. Wild type (N2) animals were reared on either control *pL4440* or *jnk-1* RNAi bacterial strains for two generations at 25 °C. Total time in sleep bouts was determined in progeny during L4/A lethargus. Note that *jnk-1* alleles alter sleep bouts, suggesting that the RNAi treatment shown here is ineffective. Validation of RNAi knockdown of *jnk-1* mRNA or protein was not undertaken. Wild type *(L4440* control *RNAi)* n = 9, wild type *(jnk-1 RNAi)* n = 15. p value < 0.5. Results reported as a box plot. Box represents the two middle quartiles, horizontal line indicates mean, and bars represent the minimum and maximum. Significance was assessed by student’s two-tailed t-test with p value < 0.5.
