# Supplementary figures and images for: Normal sleep bouts are not essential for C. elegans survival and FoxO is important for compensatory changes in sleep
Source: BMC Neurosci. 2018 Mar 9;19:10. doi: 10.1186/s12868-018-0408-1 (PMC5845181; doi:10.1186/s12868-018-0408-1)

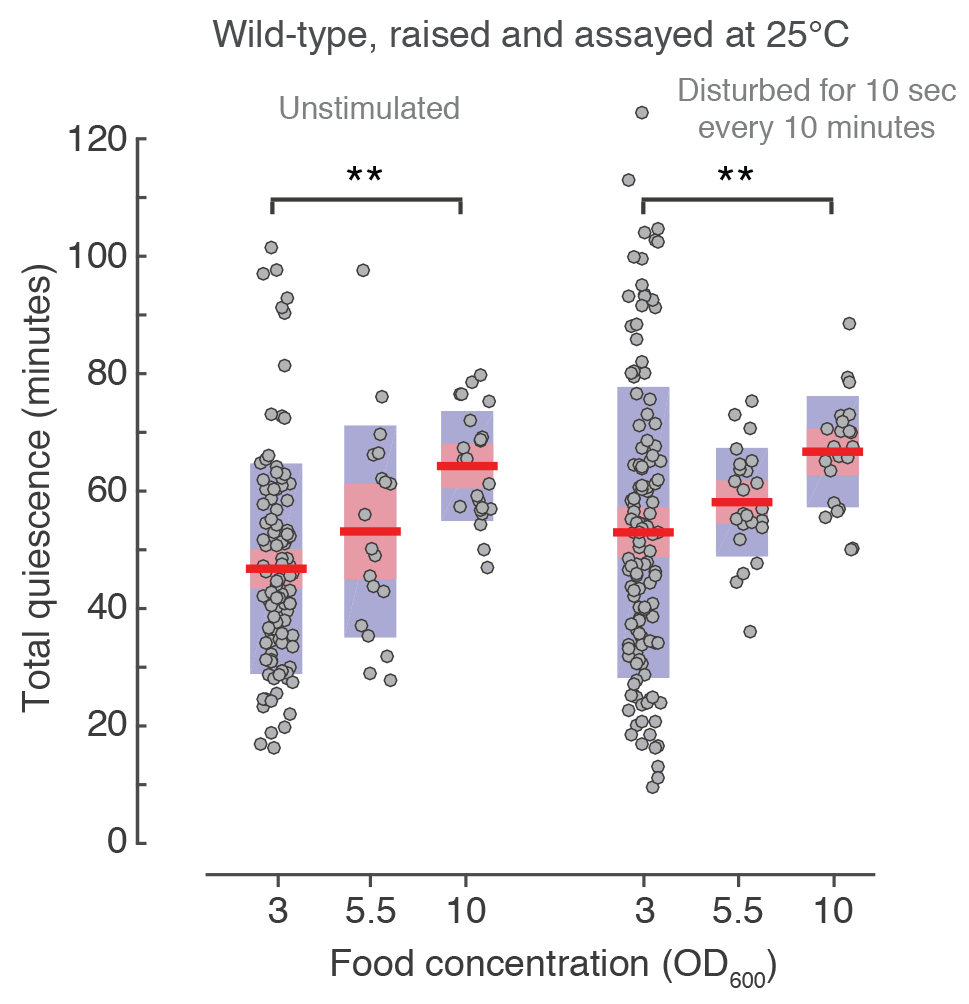

Supplement: Supplementary file 1 — Additional file 1: Food concentration alters the quantity of L4/A lethargus motionless sleep bouts. Left: the total amount in sleep bouts (sum of durations of all quiescent bouts) of undisrupted wild-type animals, raised and assayed at 25 °C. Right: the total time in sleep bouts of animals repeatedly exposed to mechanical vibrations (10 s of 1 kHz vibrations every 10 min). Excess motion caused by the mechanical stimulus was compensated for and the resulting total time in sleep bouts increased with food concentration, exhibiting a similar trend to the case of undisrupted animals. Horizontal lines, inner boxes, and outer boxes depict means, standard errors of the mean, and standard deviations, respectively. Sample sizes are noted in parentheses and double asterisks denote significant differences (p < 0.01). [file 12868_2018_408_MOESM1_ESM.tif]

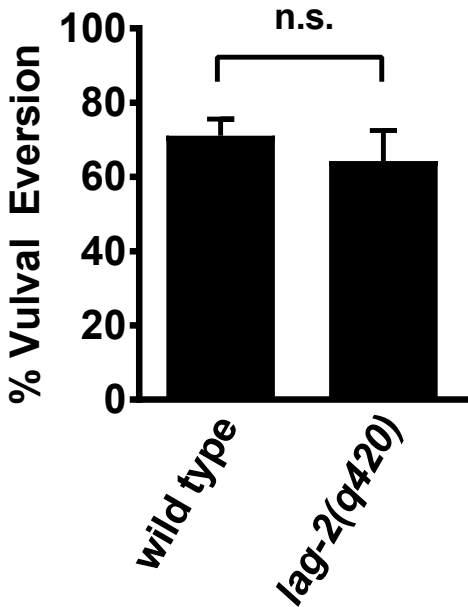

Supplement: Supplementary file 6 — Additional file 6: Decreased lag-2 function does not slow vulval development. The progeny of wild type and lag-2(q420) animals raised at 25.5 °C were selected at the L4 stage, prior to lethargus entry. Vulval eversion was scored after 3 h; the percentage of animals completing vulval eversion was recorded. Significance was assessed by student’s two-tailed t-test p value < 0.5; error bars represents SEM from 3 trials. Total number of animals: wild type n = 45 and lag-2(q420) n = 42. [file 12868_2018_408_MOESM6_ESM.pdf]

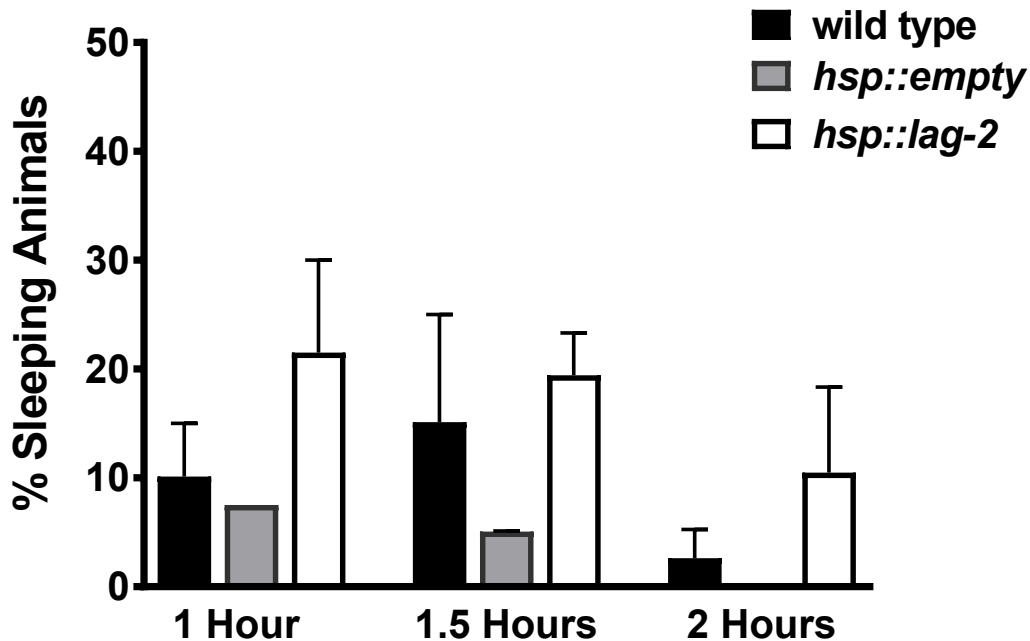

Supplement: Supplementary file 7 — Additional file 7: Increased lag-2 expression in adults induces anachronistic sleep bouts. Animals carrying hsp::empty, hsp::lag-2 cDNA transgenes, or wild type animals were heat shocked for 1.5 h at 34 °C. After heat shock, animals were allowed to recover at 20 °C for an additional 1 h to recover from stress-induced quiescence (shown in first set of columns). Sleep was scored for all genotypes within 15 min, based on the absence of feeding and movement. Inappropriate sleep in adult animals expressing hsp::lag-2cDNA transgene was reversible, disappearing by 2 h post-heat shock. For all genotypes, n = 40 animals; error bars represent the SEM from 2 independent trials. [file 12868_2018_408_MOESM7_ESM.pdf]

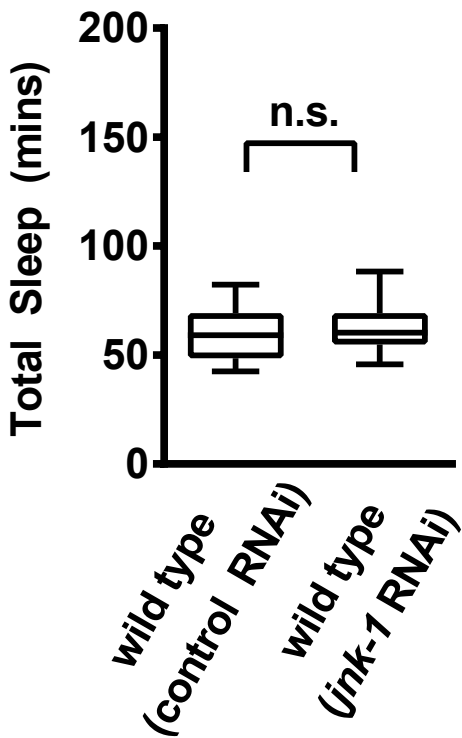

Supplement: Supplementary file 8 — Additional file 8: jnk-1 RNAi knockdown does not alter sleep bout quantity. Wild type (N2) animals were reared on either control pL4440 or jnk-1 RNAi bacterial strains for two generations at 25 °C. Total time in sleep bouts was determined in progeny during L4/A lethargus. Note that jnk-1 alleles alter sleep bouts, suggesting that the RNAi treatment shown here is ineffective. Validation of RNAi knockdown of jnk-1 mRNA or protein was not undertaken. Wild type (L4440 control RNAi) n = 9, wild type (jnk-1 RNAi) n = 15. p value < 0.5. Results reported as a box plot. Box represents the two middle quartiles, horizontal line indicates mean, and bars represent the minimum and maximum. Significance was assessed by student’s two-tailed t-test with p value < 0.5. [file 12868_2018_408_MOESM8_ESM.pdf]
