# Supplementary material for: Normal sleep bouts are not essential for C. elegans survival and FoxO is important for compensatory changes in sleep
Source: BMC Neurosci. 2018 Mar 9;19:10. doi: 10.1186/s12868-018-0408-1 (PMC5845181; doi:10.1186/s12868-018-0408-1)
Supplement: Supplementary file 2 — Additional file 2: daf-16 is not required for hypertonic stress resistance in osm-7; osm-11 animals. Hypertonic stress resistance was examined in young adult animals moved to 500mM NaCl NGM plates for 10 min. osm-7(tm2256) and osm-11(rt142) are complete loss of function alleles for Notch DOS family co-ligands. daf-16(mu86) is a partial loss of function allele. Loss of Notch DOS co-ligands results in resistance to hypertonic stress, based on inability to move, spontaneously or upon prodding, after 10 min on 500mM NaCl NGM plates. Partial loss of daf-16 function does not alter hypertonic resistance in these animals. n = 40 animals for all genotypes. [file 12868_2018_408_MOESM2_ESM.pdf]

Genotype	500mM NaCl (% Death)
wild type	100%
daf-16(mgD50)	100%
osm-7(tm2256);osm-11(rt142)	0%
daf-16(mgD50); osm-7(tm2256);osm-11(rt142)	0%
