# Supplementary material for: Normal sleep bouts are not essential for C. elegans survival and FoxO is important for compensatory changes in sleep
Source: BMC Neurosci. 2018 Mar 9;19:10. doi: 10.1186/s12868-018-0408-1 (PMC5845181; doi:10.1186/s12868-018-0408-1)
Supplement: Supplementary file 3 — Additional file 3: Simultaneous loss of daf-16 and aptf-1 does not decrease survival. Survival after L4/A lethargus is reported as percentage of animals failing to shed larval cuticle, undergo vulval eversion, and/or reach adult stage with normal locomotion and response to touch. The number of dead animals (animals not moving or feeding after prodding) was scored at 6 and 24 h after the start of L4/A lethargus. n = 75 animals for all genotypes. [file 12868_2018_408_MOESM3_ESM.pdf]

**Genotype**	**6 hours post L4 lethargus**	**24 hours post L4 lethargus**	**# Animals tested**
wild type	0%	0%	75
daf-16(mgD50)	0%	0%	75
aptf-1(gk794)	0%	0%	75
daf-16(mgD50); aptf-1(gk794)	0%	0%	75
