# Supplementary material for: Normal sleep bouts are not essential for C. elegans survival and FoxO is important for compensatory changes in sleep
Source: BMC Neurosci. 2018 Mar 9;19:10. doi: 10.1186/s12868-018-0408-1 (PMC5845181; doi:10.1186/s12868-018-0408-1)

A

Genotype	Lethargus Duration (hours)	Average Sleep Bout Duration (sec)	Average Number of Sleep Bouts	Average Number of Sleep Bouts/Hour
wild type 15°C	4.1	37	180	43
lag-2(q420) 15°C	4.3	36	172	40
wild type 25.5°C	2.9	26	120	41
lag-2(q420) 25.5°C	3.7 *	27	163 *	44

B

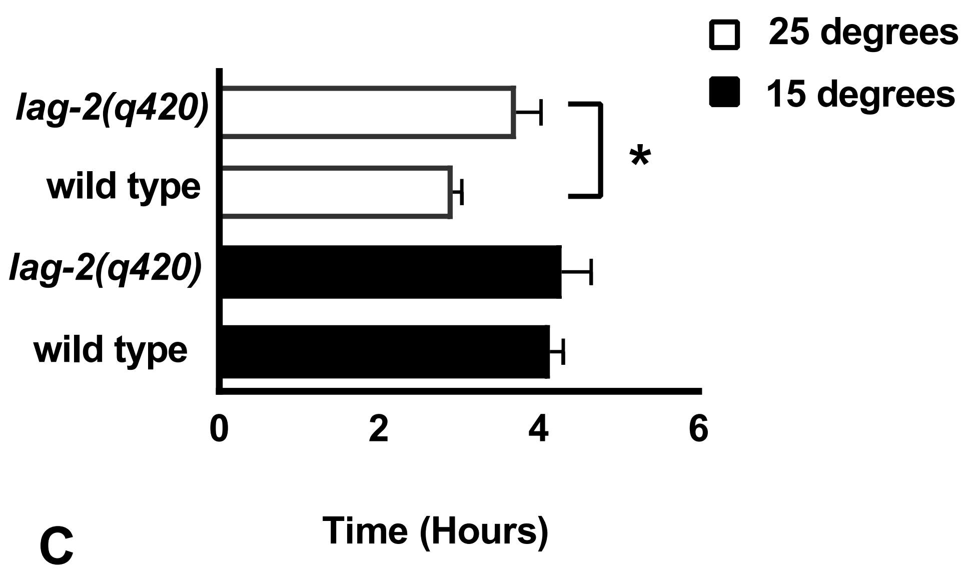

C

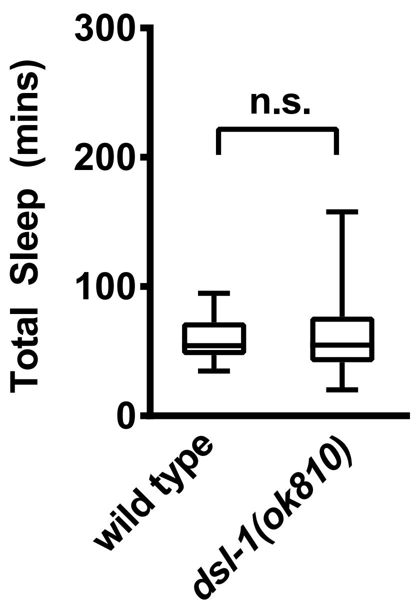

Supplement: Supplementary file 4 — Additional file 4: Notch ligands and sleep during L4/A lethargus. A) Increased time in lethargus results in increased sleep in animals with decreased lag-2 function. Detailed breakdown of sleep metrics for wild type and lag-2(q420) animals at 25.5 °C. Average sleep bout duration in seconds and average number of sleep bouts is reported per hour. Statistical significance assessed by student’s two tailed t-test; * denotes p < 0.005 for wild type versus lag-2(q420). All results available in supplemental raw data file for this and other panels. B) Partial loss of lag-2 function results in increased lethargus duration. lag-2(q420) is a temperature-sensitive, loss of function allele with decreased function at higher temperatures. Lethargus duration was assessed during L4/A lethargus, wild type or lag-2(q420) animals were shifted from 15 °C to the restrictive temperature of 25.5 °C. Total lethargus duration during L4/A lethargus were examined in F1 progeny at 25.5 °C. Lethargus duration is reported in hours; error bars indicate SEM. Note that lethargus is determined by presence/absence of motionless sleep bouts, not by morphological/developmental criteria. Statistical significance was assessed by student’s two tailed t-test; * denotes p < 0.02. At 15 °C, wild type n = 13, lag-2(q420) n = 13. At 25.5 °C wild type n = 13, lag-2(q420) n = 14. C) Loss of dsl-1 does not alter sleep. Progeny of animals were reared at 25 °C were assayed at 22 °C for total time in sleep bouts during L4/A lethargus. dsl-1(ok810) is a complete loss of function allele. Results presented as a box plot. Wild type n = 17, dsl-1(ok810) n = 16. No difference between genotypes based on student’s two tailed t-test. [file 12868_2018_408_MOESM4_ESM.pdf]
